# A focused antibody library for selecting scFvs expressed at high levels in the cytoplasm

**DOI:** 10.1186/1472-6750-7-81

**Published:** 2007-11-22

**Authors:** Pascal Philibert, Audrey Stoessel, Wei Wang, Annie-Paule Sibler, Nicole Bec, Christian Larroque, Jeffery G Saven, Jérôme Courtête, Etienne Weiss, Pierre Martineau

**Affiliations:** 1 CNRS, UMR5160, CRLC, 15, av, Charles Flahault, BP14491, 34093, Montpellier Cedex 5, France; 2 CNRS, UMR 7175, ESBS, Bld. Sébastien Brant, BP 10413, F-67412 Illkirch, France; 3 University of Pennsylvania, Department of Chemistry, 231 S. 34 Street, Philadelphia, PA 19104-6323, USA; 4 Université de Montpellier I, Montpellier, France

## Abstract

**Background:**

Intrabodies are defined as antibody molecules which are ectopically expressed inside the cell. Such intrabodies can be used to visualize or inhibit the targeted antigen in living cells. However, most antibody fragments cannot be used as intrabodies because they do not fold under the reducing conditions of the cell cytosol and nucleus.

**Results:**

We describe the construction and validation of a large synthetic human single chain antibody fragment library based on a unique framework and optimized for cytoplasmic expression. Focusing the library by mimicking the natural diversity of CDR3 loops ensured that the scFvs were fully human and functional. We show that the library is highly diverse and functional since it has been possible to isolate by phage-display several strong binders against the five proteins tested in this study, the Syk and Aurora-A protein kinases, the αβ tubulin dimer, the papillomavirus E6 protein and the core histones. Some of the selected scFvs are expressed at an exceptional high level in the bacterial cytoplasm, allowing the purification of 1 mg of active scFv from only 20 ml of culture. Finally, we show that after three rounds of selection against core histones, more than half of the selected scFvs were active when expressed *in vivo *in human cells since they were essentially localized in the nucleus.

**Conclusion:**

This new library is a promising tool not only for an easy and large-scale selection of functional intrabodies but also for the isolation of highly expressed scFvs that could be used in numerous biotechnological and therapeutic applications.

## Background

Intrabodies are defined as antibody molecules which are ectopically expressed inside the cell [1,2]. The concept of using intrabodies can result in the induction of a phenotypic knockout either by directly inhibiting the function of the targeted antigen or by diverting a protein from its normal intracellular location [3]. The main advantage of using intrabodies instead of RNA inhibition is that the inhibition is done at the protein level. As such, it is possible to target post-translational modifications or a specific conformation of the antigen [4]. In addition, by targeting antibody molecules to specific subcellular compartments using addressing signals [5], the intrabody induced phenotypic knockout can be restrained to a specific cell compartment. Altogether, this makes intrabodies a very promising tool for the study of protein function *in vivo *[6] and for the development of highly specific therapies [7].

One of the main problems associated with intrabodies is that most scFvs are not able to fold under the reducing conditions of the cell cytosol and nucleus, where most of the interesting targets are located. This is thought to be due to the limited stability of scFvs after the two conserved disulfide bonds are reduced, as occurs in the cell cytosol [8]. Indeed, *in vitro*, most of the scFvs cannot be renatured under reducing conditions [9,10]. To be an efficient intrabody a scFv must thus present a high *in vitro *stability [11]. Recent studies using either statistical analyses of scFv sequences [12] or an experimental approach [13] have shown that less than 1% of the scFvs are stable enough to be high quality intrabodies and that only about 10% have a "moderate chance" to be functional *in vivo*. In addition, even if a scFv protein is indeed stable enough in its reduced form to be expressed and active *in vivo*, other parameters such as protease susceptibility [14] or folding kinetics [10] may also influence the final *in vivo *fate of the protein and are critical for intrabody expression and activity [15].

In order to get an active intrabody it is thus usually needed to screen several clones *in vivo*, looking for the best expressed scFv with a biological activity. In order to facilitate this step, it has been proposed to first screen interesting clones using two-hybrid systems before testing them in cells [12] or even to select them directly in yeast [6]. Several very potent intrabodies have been isolated with such approaches [16] and this has allowed the isolation of several highly stable antibody frameworks [13].

As a more general strategy authors have proposed to stabilize scFvs *in vivo *using a fusion partner like the Maltose Binding Protein [17] or to construct a scFv library tailored for intracellular expression [15]. Ideally, such a library should only contain scFvs able to fold under the reducing conditions that pertain in the cell cytoplasm. Several groups have constructed antibody libraries based on a small number [18,19] or even a single framework [15,20-22]. In addition, several studies have shown that the framework stability and folding properties are at least partially conserved upon loop grafting to confer a new specificity. This is both true in the periplasm [23] and in the cytoplasm [24] of *Escherichia coli *for scFv and VHH domains [25]. These findings suggest that it may be possible to construct a scFv library based on a single optimized framework for intrabody selection.

We have recently obtained by molecular evolution a human scFv, called scFv13R4, which is expressed at high levels in *E.coli *cytoplasm [26]. This scFv is also expressed under a soluble and active conformation in yeast [27] and mammalian cells [3,5,28]. This scFv is very stable *in vitro *and can be renatured in presence of a reducing agent. In addition, analysis of its folding kinetics showed that it folds faster than the parent scFv and aggregates more slowly *in vitro *[10]. The mutations isolated are mainly located in the VH domain and seem to be highly specific to this particular scFv since they cannot be transferred to a very homologous VH domain [29]. Interestingly, most of these mutations decreased the homology between the original scFv13 sequence and the intrabody consensus sequence described by Visintin et *al*. [30] underlining again their non-general nature.

In this paper, we have constructed a human scFv library, based on the framework of the optimized scFv13R4, that contains more than a billion clones. By using optimized CDR3 loops and filtering steps to eliminate the non-expressed clones, we only retained in the library the cytoplasmically expressed scFvs without compromising the diversity, as tested with several proteins used as antigen. Most of the scFvs in the library are expressed in *E.coli *and in mammalian cytoplasm, and are functional as intrabodies. This new library is a promising tool for facile and large-scale selection of functional intrabodies.

## Results

**Figure 1:**
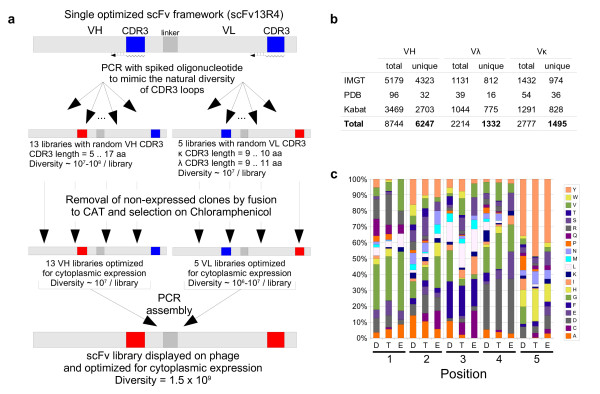
**Library construction**. (**a**) Schematic outline of the steps followed during library construction. The critical steps are: introduction of tailored CDR3 loops in an unique human scFv framework; removal of non-expressed clones by fusion with the CAT enzyme and selection on CAM plates; recombination of the 13 VH and 5 VL libraries, and display on phage. (**b**) Summary of the CDR3 loops collected in the database. (**c**) Distribution of the amino acids at each position of the 5 amino acid long VH CDR3s from 55 rearranged human antibodies (D), 43 sequenced clones from the library (E), and predicted from the oligonucleotide sequence H3_5 given in additional file 2 (T).

The aim of this project was to construct and validate a human scFv library, based on a single framework, optimized for intracellular expression and displayed on filamentous phage. To achieve this goal, the following principles have been used during library construction (Figure 1a): i) We used the antibody framework of the previously optimized scFv13R4 human scFv [26]; ii) We introduced diversity only in the CDR3 loops and mainly in the H3 region by using degenerate oligonucleotides [20]; iii) We designed the oligonucleotides so as to have the same representation of the amino acids as that observed in natural human CDR3 loops [31,32]; iv) We removed the non expressed VH and VL sequences by fusing the scFv genes to the CAT enzyme and selecting for CAM resistance [33,34]. Altogether, these principles should result in a large fully human synthetic scFv library optimized for intrabody selection.

### A database of human CDR3 sequences

We compiled human CDR3 sequences from three main sources: the Kabat database [35], the IMGT database [36] and the RCSB PDB [37]. After removing the duplicates, the database contained 6247 H3, 1332 λ CDR3 and 1495 κ CDR3 unique sequences (Figure 1b). It can be noted that most of the H3 sequences were unique since, for instance, in the Kabat database, 2703 H3 sequences (78%) were found only once among the 3469 complete H3 sequences. The result was comparable in the case of L3 and K3 since, respectively, 74% and 64% of the sequences were unique in the Kabat database. This underlines the very high variability of the human CDR3 sequences.

As noted by several authors, this variability is not evenly distributed in the loop, and the frequency of each amino acid varies from one position to another and for each loop length [18,38]. In addition, the amino acid distribution depends on the animal origin of the antibody sequence [32]. This bias can be due to a structural constraint as for instance in the case of the antepenultimate residue which is frequently an aspartate (D101 using Kabat numbering scheme) and plays an important role in the switch between the extended and the kinked conformation of the H3 [39]. In other cases, this bias may only be due to the limited number of sequences available for the D and J segments, and amino acids other than those found in natural antibodies may be tolerated [40]. For the construction of the library we decided to make CDR3s that mimic the natural distribution for two main reasons: i) we wanted the scFvs generated to be as human as possible for possible use in human therapy [41]; ii) since the antibodies found in the database are functional, maintaining their amino acid distribution will more likely result in functional antibodies, since we incorporated at each position only the amino acids tolerated in natural antibody CDRs [38].

CDR3 sequences from the database were aligned by length, and the frequency for each of the 20 possible amino acids at each position and for each loop length were calculated. In the case of the light chain CDR3s, sequences were analyzed independently for each class. In the case of the H3 sequences, this resulted in 35 tables, one for each H3 length between 1 and 34 amino acids. For a loop of length n, this table contained 20 n frequencies. This is illustrated in figure 1c with the 5 amino acid long H3 loop case (columns D). The only strongly variable position was the position 2 where the most frequent amino acid (Tyr) was only present in 16% of the loops. This is not surprising since this residue is frequently in contact with the antigen [42]. By contrast, strong biases were observed at position 1 (29% of Gly), 4 (45% of Asp and Gly) and 5 (36% of Tyr). The number of highly variable positions increased with the loop length but, in all the cases, none of the positions was truly random. For the other loop lengths, the frequencies are given in additional file 1 "Amino acid distribution in CDR3s".

### Design of the oligonucleotides encoding the CDR3 loops

Eighteen oligonucleotides were designed to follow the amino acid distribution found in the compiled CDR3 database. We used 192 optimized mixes of the four nucleotides at each position of the codon to match as well as possible the desired amino acid distribution (see [31] and references therein). Plots showing the target and the oligonucleotide-encoded distributions are given in additional file 1 "Amino acid distribution in CDR3s". The process is illustrated in figure 1c with the 5 amino acid long H3 loops. D bars represent the frequencies of the 20 amino acids in the human database at the 5 positions. T bars are the expected frequencies with the optimized mixes (after rounding the nucleotide frequencies at the nearest 5%, see Methods). Due to the constraint of the genetic code, some positions were not perfectly optimized. For instance, at position 3, alanine and glycine were under-represented in our mix and we had to introduce a substantial amount of some non naturally found amino acids like cysteine in order to match other amino acid frequencies. There was however a good overall agreement between the database and the oligonucleotide-encoded frequencies since the most frequently found amino acids were represented in the library and the rare ones were most of the time present at a low frequency. The sequences of the 18 degenerate oligonucleotides used to construct the CDR3 loops are given in additional file 2 "Sequences of the spiked oligonucleotides used to introduce the random CDR3 loops".

### Construction of the library

We constructed independent libraries for each CDR3 loop length. This was done independently for the heavy and the two classes of light chains. For each library, random CDR3 loops were introduced by PCR and the resulting library cloned back in the scFv13R4 gene fused to the CAT gene in vector pscFvΔCAT (see below). This resulted in libraries of scFv13R4 clones with one and only one randomized CDR3 loop.

We validated the approach by first constructing the 5 amino acid long H3 loop library. We sequenced 43 randomly chosen clones and the frequencies of the 20 amino acids at each of the five positions are shown in figure 1c (columns E). Some positions diverged from the expected values as, for instance, the glycine at position 2, which is over-represented in our library. However, on average, the distribution in the library matched the expected distribution. This showed that the quality of the oligonucleotides was good enough for the approach and that the resulting library followed the natural distribution of the amino acid in human H3 loops.

Using this approach, we constructed 13 VH libraries with H3 loops ranging from 5 to 17 amino acids since more than 85% of the human H3 lengths are within this range. The same strategy was used for the VL libraries. We introduced random CDR3 loops of 9 and 10 amino acids modeled on the human κ loops and ranging from 9 to 11 amino acids for those modeled on the human λ loop sequences. These loop lengths represented respectively 81% and 85% of the human CDR3 loops in the database. The 18 libraries were constructed and contained at least 3.6 × 10^6 ^clones each (Table 1).

**Table 1:** Diversity of the CDR3 libraries

		**CAM phenotype^b^**			
			
	**Initial diversity^a^**	**++**	**+**	**-**	**final diversity^c^**
H3-5	1.4e8	15/20	3/20	2/20	1.1e8
H3-6	2.7e7	20/20	0/20	0/20	2.7e7
H3-7	9.2e7	16/20	3/20	1/20	7.4e7
H3-8	5.0e6	14/20	4/20	2/20	3.5e6
H3-9	2.4e8	16/20	1/20	3/20	1.9e8
H3-10	1.0e7	10/20	6/20	4/20	5.0e6
H3-11	2.2e7	9/12	1/12	2/12	1.7e7
H3-12	1.0e7	15/20	2/20	3/20	7.5e6
H3-13	2.3e7	8/12	3/12	1/12	1.5e7
H3-14	1.0e7	12/20	4/20	4/20	6.0e6
H3-15	2.1e7	8/12	3/12	1/12	1.4e7
H3-16	1.0e7	12/20	2/20	6/20	6.0e6
H3-17	1.1e7	12/20	4/20	4/20	6.6e6
K3-9	3.6e6	11/16	1/16	4/16	2.5e6
K3-10	4.4e6	10/16	1/16	5/16	2.8e6
L3-9	1.7e7	11/20	6/20	3/20	9.4e6
L3-10	1.5e7	10/20	9/20	1/20	7.5e6
L3-11	1.8e7	15/20	3/20	2/20	1.4e7

^a ^Initial diversity of the library cloned as fusion with CAT and selected on ampicillin. This is the number of clones obtained after transformation.

^b ^Between 12 and 20 clones from the transformation were checked on CAM plates. Plates were incubated for 16 h at 37°C and colony size estimated. Columns ++,+ and - give, respectively, the fraction of clones that grew normally, gave tiny colonies, and did not grow at all.

^c ^Diversity of the libraries selected on CAM. The diversity is estimated from column "Initial Diversity" and "CAM phenotype" by assuming that the final diversity is close to (Initial Diversity) × (CAM phenotype ++). The real diversity may be a higher since some of the clones noted + in column "CAM phenotype" may be present in low abundance.

Some of these clones, however, may not be functional as a result of the cloning steps (stop codons present in the oligonucleotides and PCR) or because of a poor expression level in the bacterial cytoplasm. To remove these non functional scFvs, we selected for clones expressed in the bacterial cytoplasm by means of a fusion between the scFv and the CAT enzyme [33]. We tested different CAM concentrations for this selection step ranging from 15 to 200 μg/ml. At the highest concentration, the library was indeed enriched in well-expressed scFvs, but also in clones containing recombined plasmids harboring partial or complete deletions of the scFv gene (data not shown). We thus plated the libraries on a medium CAM concentration (30 μg/ml). This concentration was high enough to remove all the non-expressed or strongly aggregating scFvs but did not result in a detectable amount of plasmids harboring scFv deletion. The size of the libraries of expressed scFv was thus estimated as the product of the original library size (selected on ampicillin) by the frequency of the CAM^R ^clones. The sizes of the 18 libraries ranged from 2.5 × 10^6 ^to 1.9 × 10^8 ^(Table 1).

**Figure 2:**
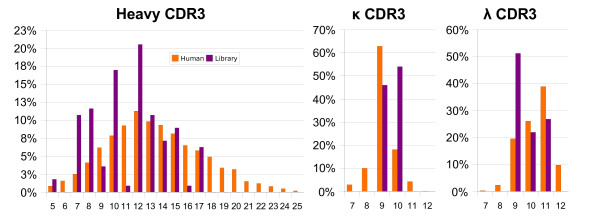
**CDR3 length distribution**. Distribution of the CDR3 lengths in the database and in 118 sequenced clones from the library (see additional file 3: Sequence of randomly picked clones).

The 18 CAM selected libraries were assembled in amounts proportional to the natural distribution of CDR3 loop lengths in human antibodies to give a final library of more than a billion of clones. Since the theoretical possible diversity is about 10^15 ^(~13 VH × 10^7 ^× 5 VL × 10^6^), it is very unlikely to obtain twice the same clone in the final library. One hundred and eighteen clones were sequenced to determine loop lengths and sequences. As shown in figure 2, all the introduced lengths except 6 for the H3 were found in the library. 11 and 16 amino acid long H3 loops were under-represented in the library. This is presumably due to the poor quality of these oligonucleotides as shown by their profile on an Agilent Bioanalizer (data not shown). H3 loop lengths ranging from 7 to 12 were over-represented in the library but only by a two-fold factor. The H3 loop lengths between 8 and 17 amino acids, which are the most frequently found in human antibodies, were all present in the library. The number of sequenced clones was too small to analyze the frequency of the amino acids found in the CDR3 loops. Except for some contaminations with the original scFv13R4 sequence (4% for H3), no CDR3 sequence was found twice in the sequenced clones. The sequences of the 118 clones are shown in the additional file 3 "Sequence of randomly picked clones".

### Cytoplasmic expression of scFvs

The idea underlying the library construction is that the CAM selection step of the VH and VL libraries should result in only expressed scFv proteins. Indeed, since only the CDR3 loops are modified between scFv13R4 and the libraries, most of the interface residues between the two domains are conserved between clones [43]. It is thus likely that any VH will assemble correctly with any VL and that the expression level of the resulting scFv will be close to that of both clones from the VH and VL libraries selected on CAM. We tested this hypothesis by picking random clones of the final library and expressing them in *E.coli *cytoplasm and in mammalian cytosol.

**Figure 3:**
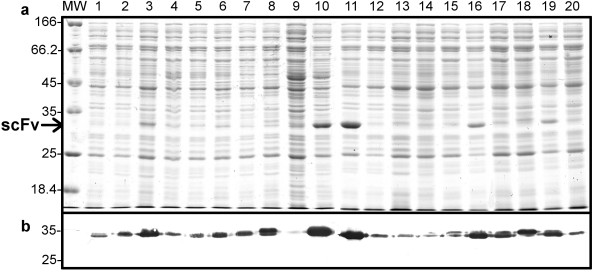
**Expression in *E. coli *cytoplasm**. Twenty clones from the library were cloned in a cytoplasmic expression vector and expressed in *E.coli *under the control of the T7 promoter. Soluble extracts were prepared, separated by SDS-PAGE, and analyzed by Coomassie staining (a) or Western-blot (b) using 9E10 and an alkaline phosphatase conjugated anti-mouse IgG antibody (substrate BCIP/NBT). Each lane corresponded to 2 × 10^7 ^cells. The arrow on the left indicates the position of the scFv.

Twenty clones were tested in *E.coli *and 19 of them showed some soluble expression in the cytoplasm (Figure 3). One-fourth of the clones (5/20; clones 3, 10, 11, 16, 19) were expressed at very high levels since the scFvs were clearly visible on a Coomassie stained gel. To obtain a more global view of the soluble expression levels in *E.coli*, the library was cloned in front of the GFPuv gene under the control of the T7 promoter. If the scFv is soluble and expressed in the cytoplasm, this should result in GFP activity that can be directly monitored on an UV transilluminator [44,45]. About 1000 clones were tested for the presence of detectable GFP activity and approximately 60% exhibited a GFP^+ ^phenotype (data not shown) showing again that most of the scFv clones from the library were expressed in *E.coli *cytoplasm.

**Figure 4:**
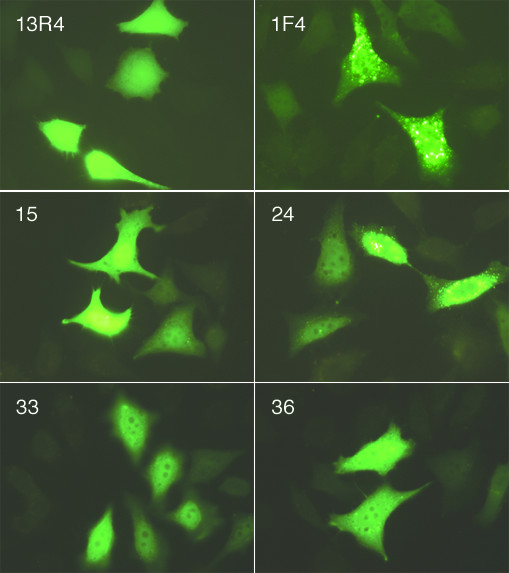
**Expression of randomly picked scFvs in HeLa cells**. Cells were transfected with scFv-EGFP constructs as indicated. 13R4 and 1F4 represent the positive and negative controls, respectively [5]. At 24 h post-transfection, cells were fixed and visualized under a fluorescent microscope with the fluorescein isothiocyanate filter set. The micrographs represent typical fields containing a similar number of cells in each case. Magnification: × 400.

We next tested the expression of the library in mammalian cells. Fifteen scFvs were cloned in a mammalian expression vector as fusions with the EGFP gene and under the control of the SV40 early promoter, then transfected in HeLa cells. Typical results are shown in figure 4. Three clones were expressed at a high soluble level, comparable to that of the parental scFv13R4 (clone 15), 10 scFvs were found to be mainly soluble but some aggregated material was still present in the cell (clones 33 and 36) and 2 clones accumulated essentially as cytoplasmic aggregates (clone 24), as observed with the hybridoma-derived anti-oncoprotein E6 scFv 1F4 (Figure 4) [28]. In conclusion, thirteen out of the fifteen scFvs tested were expressed as soluble proteins that could be easily detected in the cytoplasm and in the nucleus of the transfected cells.

Altogether these results showed that more than 85% of the clones from the final library expressed soluble scFv in *E.coli *(16/20) and mammalian cytoplasm (13/15), while about 20% of them expressed scFv at a very high level (5/20 in *E. coli *and 3/15 in eucaryotic cells). This is a great improvement over the results obtained by other authors with non optimized scFv libraries [12].

### Selection of binders

**Figure 5:**
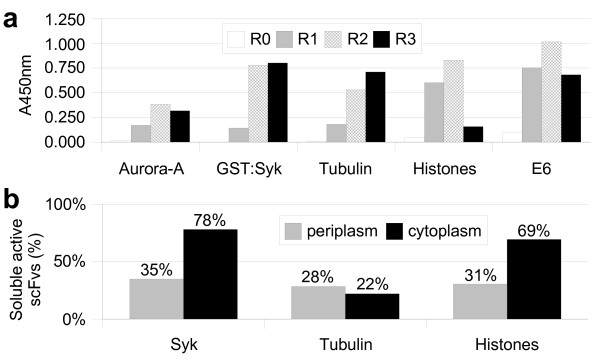
**Selection of binders against five purified proteins**. (**a**) 2.5 × 10^10 ^phages from each round of selection were tested by ELISA against their respective antigen and revealed using an anti-M13 HRP conjugated monoclonal antibody (Pharmacia). R0 is the non selected library and Rn the stock obtained after the n^th ^round of selection. Specificity was tested on BSA for the 3^rd ^rounds of selection (A450 nm ~0.1 – 0.3). (**b**) Percentage of soluble active scFvs (Absorbance > 0.1) selected after three rounds against the indicated protein and expressed either in the periplasm (gray) or in the cytoplasm (black).

As shown above, the library contains a high proportion of expressed clones but it remains to be shown that antibodies against particular proteins can be selected. We thus used the phage displayed library to select for binders against five different antigens using purified proteins adsorbed on microtiter plates. We performed three rounds of selection and the eluted phages were tested by ELISA against the immobilized antigens. As shown in figure 5a, in all the cases a positive signal was obtained after a single round of selection. This signal increased strongly after two rounds and did not increase further during the third round of selection. This very fast selection process was presumably due both to the focused library itself, which contains only expressed scFvs resulting in a low background, and to the use of a trypsin-sensitive helper phage that further decreased the background level [46,47].

We next tested whether the library contained clones expressing soluble scFvs in the periplasm. The non-suppressive HB2151 strain was infected with the phages eluted after the third round of selection against tubulin, GST:Syk and the core histones. Periplasmic extracts were prepared and tested for binding activity by ELISA (Figure 5b). In the three cases, 12–20% of the clones gave a strong signal with absorbance values higher than 0.5 (10 times the background) and about 30% were clearly positive with an absorbance value higher than 0.1 (twice the background). These results compared favorably with those reported with other scFv libraries, underlining again the high proportion of well-expressed clones present in the library. In addition, this showed that our CAT fusion approach selected efficiently for constructs without stop codons present in the oligonucleotides. This is indeed of premium importance to isolate soluble scFvs from phage-displayed libraries since amber stop codons in CDRs are frequently selected during panning of synthetic and semi-synthetic libraries [48].

In both the previous characterizations, the scFvs were expressed under oxidizing conditions in *E.coli *periplasm, either as scFv-pIII fusion or as soluble protein. In addition, panning was done on phage, again under oxidizing conditions. We could not exclude that the selection and the screen introduced a bias towards clones expressed in the periplasm at the expense of those expressed in the cytoplasm. To test that the selected scFvs were indeed also expressed in the cytoplasm, we subcloned the same pool of clones (round 3) in a cytoplasmic expression vector under the control of the strong T7 promoter. For each antigen, ninety-five clones were tested by ELISA for binding to their respective antigen. In each case, the number of positive clones was comparable or even better than in the periplasmic screen (Figure 5b). For instance, in the case of GST:Syk, 80% of the tested clones were positive after three rounds of selection. This demonstrated that the periplasmic selection step did not decrease the proportion of soluble scFvs in the cytoplasm and that by using our library it is not necessary to directly select within the cytoplasm to avoid introducing a bias during the selection [13].

We sequenced individual clones from the 2^nd ^and the 3^rd ^round of selection against tubulin (Table 2). Most of the clones were different since only one clone from the 2^nd ^round and one from the 3^rd ^round were found twice. This demonstrated that a high diversity is still present after 3 rounds of selection. Eight of the anti-tubulin scFvs were purified by affinity chromatography from the cytoplasm. In all cases, more than 8 mg of scFv was purified from one liter of cells grown in a flask (OD_600 _= 5), and some scFvs were expressed at a level per cell comparable to the exceptionally high expression level reported for an anti-HER2 in *E. coli *periplasm (Table 2) [49]. This expression level corresponds to about 1/4^th ^of that of the original scFv13R4 but this may be presumably increased by using optimized protocols.

**Table 2:** Sequences of some anti-tubulin scFvs

	**VH CDR3**		**VL CDR3**					
						
**Name**	**Sequence**	**length**	**Sequence**	**length**	**frequency^a^**	**yield^b^**	**WB^c^**	**IF^d^**
*Round 2*								
**C12C**	SSITIFGGGMDV	12	HSREVHRTF	9	1/5	19		
**E12C**	SGGNTFDY	8	QQYYRKPWT	9	1/5	53		
**F1C**	GNADGGENWELFDK	14	QLYQNTLWT	9	2/5	52		
**H6C**	SSITIFGGGMDV	12	QQNWTSPLS	9	1/5	nd		

*Round 3*								
**2C1C**	RGRDY	5	QQYNTSPFS	9	1/6	8.6	+	-
**2E11C**	GRNVLNY	7	QQNSSSPRFT	10	2/6	8.7	+	-
**2F12C**	GRRALGN	7	QQYNTSPFS	9	1/6	45	+	+
**2G4C**	GRRALGN	7	LTWSMRSAI	9	1/6	15	+	+
**2G9C**	GRRALGN	7	LTTENSVYRLV	11	1/6	50	+	-

Sequences of the CDR3s of the best positive clones in an ELISA using cytoplasmically expressed scFv from the 2^nd ^(5 clones) and the 3^rd ^(6 clones) round of selection (Table 2). ^a ^Frequency of apparition of the scFv among sequenced clones of the same round. ^b ^Yield: mg of scFv purified from 1 liter of cells grown in a flask (OD_600 _= 5). ^c ^WB: detection of tubulin in brain extracts by Western blot. ^d ^IF: + means that the scFv is able to reveal microtubule network by Immunofluorescence (Fig. 8). The sequences of the clones 2C1C, 2E11C, 2F12C, 2G4C and 2G9C have been submitted to the EMBL database and their accession numbers are respectively AM886280, AM886281, AM886282, AM886283 and AM886284.

### Functionality of isolated scFvs as intrabodies

**Figure 6:**
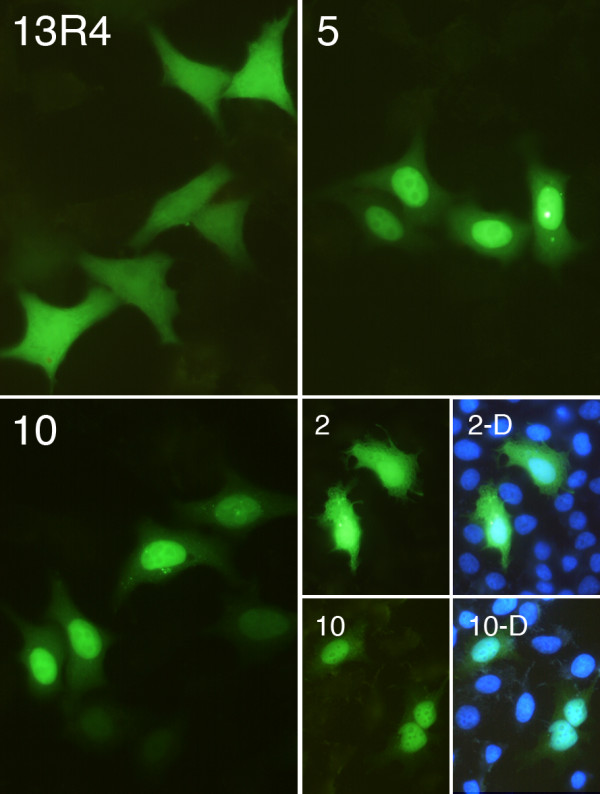
**Expression of anti-histones scFvs fused to EGFP in HeLa cells**. The cells were transfected and treated as indicated in the legend of figure 4. The pictures represent typical cells transfected with scFv13R4 and three representative anti-histones clones (2, 5 and 10). D, DAPI staining (blue) merged with the GFP signal.

To determine if the isolated scFvs were able to bind to their target *in vivo *we characterized the anti-histones scFvs expressed in human cells. The third round of selection was cloned in vector p513-EGFP and ten randomly chosen clones were transfected in HeLa cells. Typical results of the cells expressing the scFv-EGFP fusions and observed by fluorescence microscopy are shown in figure 6. One scFv was expressed as cytoplasmic aggregates. Four scFvs were expressed as soluble cytoplasmic proteins, as judged by the homogeneous staining of the cells, at a level comparable to that of the scFv13R4. Finally, three scFvs gave rise to a strong staining of the nucleus (clones 2, 6 and 9) and, two scFvs were exclusively localized in the nucleus (clones 5 and 10). Since these scFvs fused to the EGFP were expressed in the cytoplasm of the cell and did not contain a nuclear localization signal, this suggested that they were able to interact *in vivo *with the histones and were thus active inside the cell [5]. This analysis showed that about half of the clones present after the third round of selection against core histones were able to bind to their nuclear target *in vivo*. This was confirmed *in vitro *by western and dot blot with purified scFv (additional file 4 "*In vitro *characterization of some anti-histones scFvs"). In addition, sequencing of the five positive clones showed that they contained different heavy and light chain CDR3 regions (additional file 4 "*In vitro *characterization of some anti-histones scFvs").

### *In vitro *characterization of anti-tubulin scFvs

**Figure 7:**
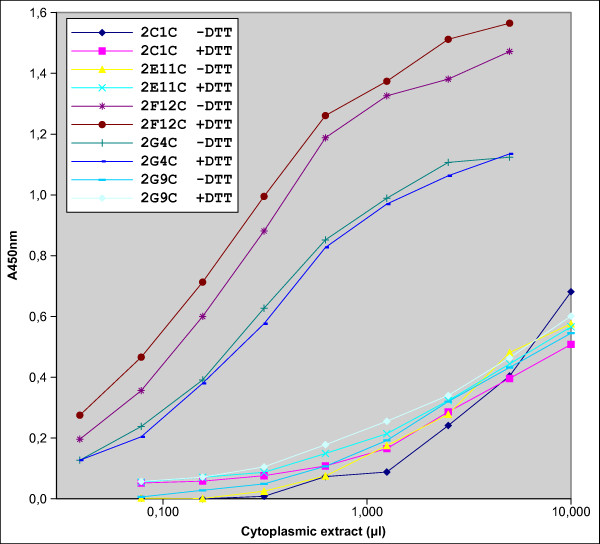
**Activity of scFvs under reducing conditions**. Extracts of cells expressing the scFvs in the cytoplasm were prepared as in figure 3 in presence or absence of 10 mM DTT. ELISA was performed in presence or absence of DTT, accordingly. x-axis: amount of extract per well. y-axis: ELISA signal at 450 nm.

To demonstrate the activity of the anti-tubulin scFv under the reducing conditions that pertain in the cell cytoplasm, we extracted the scFvs in presence of a reducing agent and we compared the ELISA signal with that obtained with the scFvs extracted under oxidizing conditions. As shown in figure 7, the five scFvs gave the same ELISA signal in both conditions, demonstrating the full activity of the scFvs under reducing conditions and thus in absence of disulfide bond formation.

**Figure 8:**
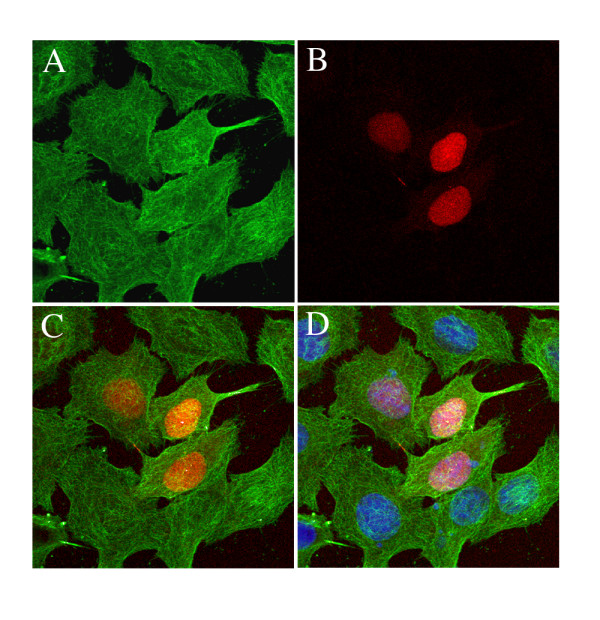
**Double staining of HeLa cells using an intrabody and by immunofluorescence**. HeLa cells were transfected with anti-histones clone 5 (Figure 6) fused to the dsRed-monomer GFP. Microtubules were revealed in the transfected cells by IF using the anti-tubulin scFv 2F12C (Table 2). Cells were observed at the appropriate wavelength to visualize: (A) 2F12C scFv (microtubules alone); (B) clone 5 intrabody (histones alone); (C) 2F12C and clone 5 (both microtubules and histones); (D) as C plus nucleus staining with DAPI.

The five scFvs were able to recognize unfolded tubulin by western blot in brain extracts and the native protein in a competition ELISA (data not shown). Finally, we tested their ability to interact with microtubules in cells by IF. Clones 2F12C and 2G4C, but no the three other scFvs, were able to reveal the microtubule network in cells. In figure 8 is illustrated the utility of this new library as a source for both *in vivo *and *in vivo *proteomic studies: HeLa cells were transfected with the anti-histones clone 5 fused to a Red-GFP then the microtubule network was revealed by IF using the 2F12C scFv.

Finally, we characterized clone 2F12C by Biacore since it was the best binder in ELISA and IF. Data were fitted to a two-state reaction model giving a Kd of 50 nM (additional file 5 "Biacore analysis of clone 2F12C"). This value is comparable to those obtained for the best scFvs obtained with other single framework libraries with variability restrained to the CDR3 loops [20,22].

Altogether, our results show that the library described in this report is highly diverse and functional and allows fast and easy isolation of *in vivo *and *in vitro *active fully human intrabodies.

## Discussion

As noted by several authors [6], despite very interesting applications in the proteomic [19] and therapeutic fields [7], intracellular immunization remains a difficult approach, and its full potential in the post-genomic area remains to be demonstrated. The usual approach to obtain efficient intrabodies requires two successive steps. First, a panel of antibodies against the target antigen must be isolated. Due to the availability of very high quality naive antibody libraries displayed on phage, this step is now easily accomplished by phage-display and can be automated in order to isolate binders against several proteins in parallel [50]. In a second step, these antibody fragments (scFv or Fab) must be tested *in vivo *for their ability to inhibit their target. However, most scFvs do not fold properly under the reducing conditions that pertain in the cytosol and the nucleus of the cell where most of the interesting targets are located. This results in the formation of aggregated and inactive scFvs, unable to interact with their target. This makes the process of identifying intrabodies from regular scFv libraries a difficult procedure even when the screening is done *in vivo *using two-hybrid system [12]. In addition, this low proportion of active scFvs in the current libraries makes the isolation of intrabodies against different epitopes of the same protein at best difficult if not impossible. More recently, to avoid this two-step procedure, Visintin and collaborators [6] demonstrated that it is possible to directly select, in a single step, efficient intrabodies in yeast using the two-hybrid system. However, the relatively low efficiency of yeast transformation restrained the size of the library to 10^7^.

In this report we describe the construction and use of a large phage displayed library of scFvs optimized for intracellular expression. The library was constructed on a single antibody framework previously evolved to improve its activity inside the cytoplasm [26]. The parental scFv is very stable, has favorable folding and aggregation kinetics [10] and is expressed at very high levels in all tested cell types [5,26,27]. Having a single framework for the construction of a library should allow more comparable expression levels between clones since most of their sequences are conserved. In addition, because CDR sequences play also a role in scFv folding and expression, we anticipated that the expression level of the clones would still exhibit some variability. To minimize these differences, we only introduced variability in the CDR3 loops for three main reasons: these loops are the most variable in antibodies and are thus more likely to be highly tolerant to sequence and length variations; the parental scFv had gained mutations in the CDR2 loops during its evolution process and we did not want to reverse back these mutations; it has been previously shown that introduction of variability in the CDR3s was enough to generate antibodies against most proteins [19]. In addition, the frequencies of the amino acids in these loops were carefully biased so as to recover the distribution observed among natural human sequences. When the expression levels of randomly picked clones were compared in the cytoplasm, despite some clear differences, a high proportion of them were correctly expressed at high levels both in *E.coli *and in mammalian cells (Figures 3 &4).

It must be noted that the design of the library allowed in the VL not only the introduction of CDR3s corresponding to the original λ light chain class but also to the κ class. Such a hybrid VL domain with full binding properties has been previously realized by grafting λ CDRs on a κ framework [11], and we have also successfully grafted the CDRs from a mouse κ scFv directed against the human papilloma virus E6 protein [44] on the scFv13R4 λ framework (manuscript in preparation). This is the case of the best isolated anti-tubulin 2F12C scFv since it contains a κ CDR3 close to the CDR3 sequence encoded by human germline IGKV1-16 [36]. This showed that such grafts can produce functional scFv and that the differences in the sequences of the CDR3 loops between the λ and the κ classes are due to evolutionary divergences and not to structural constraints.

A frequent concern when constructing scFv libraries is the simultaneous optimization of the library's diversity and size. Indeed, the size of such a library is limited by the transformation efficiency to about 10^10 ^clones. Given this "limited" number of clones, it is thus of premium importance to avoid non-expressed scFvs or duplicates. To solve this problem we used a two-step procedure. First, we constructed 18 "small" libraries for each CDR3 length (13 VH and 5 VL) and removed from them all the non-expressed clones by fusion to the CAT enzyme, in *E.coli *cytoplasm, and selection on CAM plates. This step reduced each library diversity by about 10–30%. In a second step we recombined the selected VH and VL libraries at random to generate the final diversity. We made the assumption that if a VH and a VL were expressed when associated respectively with the VL13R4 and the VH13R4, the scFv constituted with these VH and VL would also be expressed, resulting in a library containing only expressed scFvs. As shown in figure 3 this is indeed the case since 19 out of 20 clones picked at random were expressed at least partially under a soluble form in *E.coli *cytoplasm. Since this selection step was done early during the library construction, the diversity of the final library was only limited by the final transformation. In addition, this recombination step, by generating a high diversity, ensured that all the clones were unique in the final library. Altogether, this approach resulted in a library of 1.5 billion expressed scFvs.

Successful use of scFvs as intrabodies on a large scale requires several essential points to be fulfilled by the library. First, the scFv must be easy to isolate. This is the case here since we were able to isolate binders against all the tested proteins (Figure 5a). Second, the scFv should be able to fold in all the cell compartments, particularly in the reducing ones. Again, this is the case for the scFv library described herein, since more than 80% of the tested clones are at least partially soluble in the cell (Figure 3 &4). In addition, we have shown that good cytoplasmic binders can be obtained from the phage selected scFvs in *E.coli *(Figure 5b) and in eukaryotic cells (Figure 6). Finally, to get active intrabodies it is important to be able to target any epitope of a protein. It is know that the immune system is able to raise antibodies against essentially any part of the surface of a protein [51], and it remains to be proved that this is also the case with our and other phage displayed libraries. However, the high length diversity introduced in the CDR3 loops should favor a broad diversity of paratope shapes [52] and thus of the epitopes recognized.

Even if the library has been tailored for the isolation of intrabodies, it can also be used as a general purpose library to select scFvs for diagnostic and therapeutic applications. Because we designed the CDR3 diversity using expressed human sequences, the scFvs present in the library are fully human and should not induce an anti-scFv antibody response in patients [41]. However, for such applications, the affinity of the scFv for its target must be very high in order to get a good sensitivity in a diagnostic test or a strong *in vivo *effect in therapy. Since our library is based on a single framework, it should be fairly easy to improve the affinity of a selected scFv by using, for instance, chain shuffling [53], error-prone mutagenesis [54] or by optimization of the CDR1 and CDR2 loops [20].

## Conclusion

This new library is a promising tool not only for an easy and large-scale selection of functional intrabodies but also for the isolation of highly expressed scFvs that could be used in numerous biotechnological applications. The availability of scFvs based on a highly stable framework [10] that are obtainable from *E. coli *in milligram quantities from a 10 ml culture (Table 2) would be very useful for developing antibody arrays. Because of the human origin of both the framework and binding site regions [41], scFvs isolated from the library should also have therapeutic applications.

## Methods

### Bacterial strains, chemicals and enzymes

LB and 2xYT media were previously described [55]. Strain C-Max5αF' (Bio-rad laboratories) is *E.coli *K-12, [F' *lacI*^q ^*Tn*10] φ80d*lacZ*ΔM15 Δ(*lacZYA-argF*)U169 *recA*1 *endA*1 *hsdR*17(r_k_^- ^m_k_^+^) *phoA supE*44 λ- *thi*-1 *gyrA*96 *relA*1. MC1061 (ATCC #53338) is *E.coli *K-12, F- λ- *hsdR*2 *hsdM*+ *hsdS*+ *mcrA mcrB*1 *araD*139 Δ(*ara-leu*)7696 Δ(*lacIPOZY*)X74 *galE*15 *galU galK*16 *rpsL thi*. Non-suppressor strain HB2151 is *E. coli *K-12 [F'*proA*+*B*+*lacI*q *lacZ*ΔM15] *ara *Δ*(lac-pro) thi*. Chemicals were purchased from Sigma. Restriction enzymes and cloned *Taq *polymerase were from Fermentas. ProofStart and *Pfu *DNA polymerases were respectively purchased from Qiagen and Promega. Plasmid DNA, PCR and agarose separated DNA were purified using Macherey-Nagel Nucleospin kits.

### Oligonucleotides

The sequences of the 18 spiked oligonucleotides used to introduce degenerate CDR3 loops and listed in the additional file 2 "Sequences of the spiked oligonucleotides used to introduce the random CDR3 loops" have been synthesized and HPLC purified by IBA GmbH (Goettingen, Germany). Other oligonucleotides were synthesized by MWG:

T7.back CCGGATATAGTTCCTCCTTT;

T7.for CTGCTAACCAGTAAGGCAAC;

M13rev-49 GAGCGGATAACAATTTCACACAGG;

M13uni-43 AGGGTTTTCCCAGTCACGACGTT;

scFvCAT.rev AACGGTGGTATATCCAGTGA;

scFvCAT2.rev CGGTGGTATATCCAGTGATTTTT;

PliaisonH3 TGGGGCAGAGGCACCCT;

PliaisonH3.back AGGGTGCCTCTGCCCCA;

PliaisonL3 GCAGTAATAATCAGCCTCGTCC

### Plasmids

Phagemid vector pCANTAB6 [56] was used for N-terminal fusion of *Nco*I/*Not*I-scFv fragments to the minor coat protein pIII of filamentous phage M13. This phagemid is derived from pUC119 and contains in the following order: a *lac *promoter, the *pel*B leader sequence, *Nco*I and *Not*I sites for scFv cloning, a His6 and a *c*-myc tag recognized by the 9E10 monoclonal antibody [57], an amber codon and the pIII gene sequence.

For cytoplasmic expression of the scFvs in *E.coli *we used plasmid pET23NN [58]. This plasmid is derived from pET23d(+) (Novagen) and contains a T7 promoter, followed by a *Nco*I site containing the ATG initiator, a *Not*I site followed by a *c*-myc and a His6 tag.

Plasmid pscFvΔCAT is derived from pTrc99A and contains a *tac *promoter, followed by a *Nco*I site containing the ATG initiator of an out-of-frame scFv, a *Not*I site followed by the CAT gene. When a scFv is inserted within the *Nco*I-*Not*I sites, the scFv is expressed as a fusion with the CAT protein. The construction was done as following. First, the unique *BstE*II site of pTrc99A (A13038) was removed by digestion followed by 5' overhang fill-in, to form blunt ends, and ligation. The resulting plasmid was digested with *Nco*I and *Not*I, and the 4210 bp fragment purified (fragment I). Second, the unique *Nco*I site of plasmid pACYC184 (X06403) located within the CAT gene was removed by site directed mutagenesis by changing the Thr172 codon from ACC to ACG. Then the CAT gene was amplified by PCR using CAT-NotI.for (TAAGGCGGCCGCA***ATGGAGAAAAAAATCACTG) ***and CAT-HindIII.back (ACTGCCTTAAAAAGC***TTACGCC***). In the oligonucleotide sequences, the introduced restriction sites are underlined and the beginning and the end of the CAT gene are in bold-italic. The 660 bp PCR fragment was digested by *Not*I and *Hind*III, and purified (fragment II). Third, a 750 bp *Nco*I-*Not*I scFv13E6 fragment, a grafted version of the scFv13R4 containing the CDR loops of an anti-E6 monoclonal antibody (Philibert *et al*., to be published), was purified (fragment III). Four, the three fragments I, II and III were ligated to give plasmid pscFvCAT. Finally, an internal deletion of 165 bp was introduced in the scFv by removing the fragment between the two *Pst*I sites of the gene. The resulting plasmid, called pscFvΔCAT, is Amp^R ^and CAM^S ^since the deletion of the *Pst*I fragment resulted in a frameshift in the scFv.

Plasmid p513-EGFP is a derivative of pSG5 [59] and harbors the EGFP coding region (Clontech) under the control of the SV40 early promoter. The p513-scFv-EGFP constructs correspond to in frame fusions of the scFvs and the EGFP coding region with a linker of 10 residues. The scFv coding regions were amplified with oligonucleotide primers 5'-ACTGATAAGCTTGCCACCATGGCCGAGGTGC and 5'-TTGATTACTAGTGAGTTTTTGTTCTGCGGCC and inserted into the *Hind*III-*Spe*I digested p513-EGFP vector.

### Database of CDR3 sequences

We used the release 5 (August, 1992) of the Kabat database [35]. This dataset contained 44990 sequences. We extracted the 4643 human VH sequences which were not a pseudogene and were not humanized. H3 sequences were then extracted from this dataset taking first into account the nucleotide sequence when present then the amino acid sequence. Finally we kept the 3469 complete H3 sequences that contained only the 20 regular amino acids, among which 2703 were unique. The same procedure was followed for λ and κ light chains, respectively, resulting in 1044 and 1291 sequences from which 775 and 828 were unique.

We also extracted CDR3 sequences from the IMGT/LIGM-DB database the 27 Nov. 2003 [36]. We considered only the "productive/regular/human/cDNA+RNA/Rearranged" genes. We obtained 5179 H3, 1432 K3 and 1131 L3 sequences from which 4323, 974 and 812 were unique.

We collected 127 additional human antibody sequences from the Protein data bank [37]. For this we used the file of 510 sequences already compiled by Andrew Martin the 19 August 2003 [60]. The complete list of CDR3 sequences is available on request to PM.

### Spiked oligonucleotide design

In biasing the representations of the amino acids, optimized mixtures of the nucleotides at each of the three codon positions were calculated as described previously [31,61]. Premature termination of protein sequences was limited by imposing and upper bound of 0.05 on the probability of realizing a stop codon. For the 34 positions which did not satisfactorily recover the desired probabilities of the amino acids, a second optimization was done with the same method but with no constraint on the stop codon frequency. For oligonucleotide synthesis, the calculated frequencies were rounded in increments of 5% as follows: all the frequencies between 0% and 5% were rounded to 5%; other frequencies were rounded to the nearest 5%; if the resulting sum was higher than 100%, 5% was removed from the rounded amino acid frequency larger than 5% for which the difference between the rounded and the target frequency was maximal and the process iterated until the sum was 100%; if the sum was lower than 100%, 5% was added to the rounded frequency lower than 95%, for which the difference between the rounded and the target frequency was maximal and the process iterated until the sum was 100%. The sequences of the oligonucleotides are given in the additional file 2 "Sequences of the spiked oligonucleotides used to introduce the random CDR3 loops".

### Construction of VH and VL libraries

Variable CDR3 sequences were introduced in scFv13R4 by PCR assembly using a hot-start proofreading polymerase (ProofStart, Qiagen) using as template plasmid pAB1-scFv13R4p [10]. To introduce random H3 loops, the 5' of the gene with the random H3 sequence was obtained with oligonucleotides M13rev-49 and one of the 13 degenerate oligonucleotides (H3_n, see additional file 2: Sequences of the spiked oligonucleotides used to introduce the random CDR3 loops) and the 3' with PliaisonH3 and M13uni-43 (both for 20 cycles at 55°C). The two purified bands were thus assembled by PCR (30 cycles, 55°C) using M13rev-49 and M13uni-43. The resulting PCR was purified using a commercial kit (Nucleospin, Macherey-Nagel), digested for 16 h at 37°C with *Nco*I and *Not*I enzymes, then purified on gel. The same procedure was followed to introduce random L3 and K3 loops except that the pairs of primers were M13rev-49/PliaisonL3 for the 5' and one of the degenerate oligonucleotide encoding the L3/K3 loop (K3_n or L3_n, see oligo.txt) with M13uni-43 for the 3' part of the gene.

Each digested band was ligated for 16 h at 16°C with 1 μg of *Nco*I-*Not*I digested and purified pscFvΔCAT in 100 μl using 10 Weiss units of T4 DNA ligase. The ligation was heat inactivated and purified using a commercial kit (Nucleospin). The ligation was then electroporated in 300 μl of MC1061 competent cells [62] and plated on a 600 cm^2 ^square plate of LB containing 100 μg/ml of ampicillin and incubated for 16 h at 37°C. The 21 libraries (13 VH and 5 VL) were scrapped in 10 ml of LB with 10% glycerol and 10^9 ^bacteria were immediately plated on a 600 cm^2 ^square plate of LB containing 100 μg/ml of ampicillin, 1 mM IPTG and 30 μg/ml of CAM and incubated for 16 h at 37°C. The 21 libraries were scrapped in 10 ml of LB with 10% glycerol and frozen at -80°C. An aliquot was used to prepare DNA for the library assembly.

### Library assembly

The 13 VH libraries were amplified using primers M13rev-49/PliaisonH3.back using Pfu polymerase and the 5 VL libraries using scFvCAT.rev/H3_Liaison (30 cycles, 55°C). The 21 PCR bands were purified then carefully quantified on gel using ImageJ software [63]. The 13 VH bands were pooled in amounts proportional to their frequency in human H3. This mix is called VHpool. The 2 VL κ bands were pooled in order to obtain 75% of 9 amino acids and 25% of 10 amino acids loops. The VL λ bands were pooled to obtain 30% of 9, 30% of 10 and 40% of 11 amino acids loops. Finally the κ and λ mixes were pooled in order to get 50% of each class in the final mix called VLpool.

VHpool and VLpool were assembled by PCR using *Taq *DNA polymerase and primers M13rev-49/scFvCAT2.rev in 500 μl (30 cycles, 55°C). The PCR was successively digested with 20 units of *Nco*I and *Not*I for at least 6 h each, purified then quantified on gel. 50 μg of vector pCANTAB6 was successively digested with 80 units of *Nco*I and *Not*I for at least 6 h each, purified then quantified on gel. 5 μg of linearized pCANTAB6 was ligated with an equal molar amount of insert (0.84 μg) in 500 μl at 16°C using 50 Weiss units of T4 DNA ligase. The ligation was heat inactivated and purified using a commercial kit (Nucleospin). The purified ligation was then electroporated in 10 × 300 μl of C-Max5αF' competent cells [62], plated on ten 600 cm^2 ^square plate of LB containing 1% of glucose and 100 μg/ml of ampicillin. After incubation for 16 h at 37°C, cells were scrapped in 2xYT containing 10% of glycerol and kept frozen at -80°C in aliquots corresponding to twenty times the diversity.

### Antigens

Aurora-A is an His-tagged protein produced in *E.coli*. GST:Syk was expressed in *E.coli *and was a gift from P. Dariavach [64]. E6 protein from papillomavirus HPV16 was expressed in cyanobacterium Anabaena (Desplancq *et al*., to be published). Histones (a mix of H2a, H2b, H3 and H4) were purchased from Sigma (type II-AS. #H7755). Tubulin was purified from pig brain [65].

### Library rescue and selection

Library rescue was done essentially as previously described using a trypsin sensitive helper phage [66]. Briefly, an aliquot of the library corresponding to a 10 to 20 fold excess over the diversity (2–3 × 10^10 ^bacteria) was inoculated in 1000 ml of 2xYT containing 100 μg/ml ampicillin and 1% glucose and grown with shaking at 37°C until OD_600*nm *_was 0.7. 200 ml (~3 × 10^10^ cells) were infected with 5 × 10^11^ helper phage KM13 [66] and incubated without shaking for 30 min at 37°C. Cells were pelleted, resuspended in 1000 ml of 2xYT containing 100 μg/ml ampicillin and 25 μg/ml kanamycin and incubated overnight with vigorous shaking at 30°C. The supernatant containing phages was precipitated twice by adding 1/5^th ^of the volume of PEG-8000 20%, NaCl 2.5 M, resuspended in PBS supplemented with 15% of glycerol and aliquots containing 10^11^-10^12 ^phages were stored at -80°C.

To select for binders, 100 μl of purified antigens were coated in a Nunc Maxisorp 96-well plate. For the first round we used an antigen concentration of 10–100 μg/ml and 1–10 μg/ml for the subsequent rounds. Plate was washed 3 times with PBS containing 0.1% of Tween20 (PBST) and saturated 2 h at RT with PBS containing 2% of non fat milk (MBPS). 10^11^-10^12 ^phages were added per well in 2% MPBS and incubated for 2 h at RT. The plate was washed 20 times (first round) or 40 times (2^nd ^and 3^rd ^rounds) with PBST then 3 times with PBS. Excess PBS was removed and the phages were eluted by adding 100 μl of 100 mM triethylamine for 10 min at RT. The eluted phage suspension was neutralized with 50 μl of 1 M Tris-HCl pH 7.4, then digested 15 min at RT with trypsin by adding 1.5 μl of 0.1 M CaCl_2 _and 15 μl of 10 mg/ml TPCK-treated trypsin (Sigma). 1 ml of a 37°C exponentially growing Cmax5αF' strain in 2xYT was infected with 40 μl of trypsin-treated phages, incubated 30 min at 37°C without shaking, then plated on a 15 cm round Petri dish (LB, 100 μg/ml ampicillin, 1% glucose). After overnight incubation at 37°C, bacteria were recovered from the plate and used to prepare a new stock of phages using KM13 helper phage. 10^11^-10^12 ^phages of this stock were used for the next round of selection.

### Periplasmic and cytoplasmic screening

For periplasmic screening, phages from round 3 were used to infect the non-suppressive strain HB2151. Individual clones were tested for scFv expression by ELISA on antigen-coated 96-well microtiter plates as described [67]. For cytoplasmic screening, plasmid was prepared from the pool of bacteria of the 2^nd ^or 3^rd ^selection round, digested with *Nco*I and *Not*I enzymes, and the 750 bp band was cloned in *Nco*I-*Not*I digested and dephosphorylated plasmid pET23NN. Ligation was transformed in C-Max5αF', plated on LB with 100 μg/ml ampicillin and incubated for 16 h at 37°C. Cells were scrapped and the plasmid DNA prepared and used to transform chemically competent BL21(DE3)pLysS. Individual clones were grown in a 96-well microtiter plate containing 100 μl of 2xYT, 100 μg/ml of ampicillin with vigorous shaking at 37°C until OD_600*nm *_reached 0.6. IPTG was added to 0.4 mM final and the microtiter plate incubated for 16 h at 24–30°C with vigorous shaking in a humidified atmosphere. After centrifugation, cells were resuspended in 100 μl of 50 mM Tris-HCl pH7.5, 5 mM EDTA, freeze/thawed and incubated 1 h on ice. MgCl_2 _was added up to 10 mM and the DNA was digested with 10 μg/ml of DNAseI. 5–20 μl were used in an ELISA on an antigen-coated 96-well microtiter plate (Nunc Maxisorp). Revelation was done using 9E10 monoclonal antibody followed by an HRP conjugated anti-mouse IgG antibody.

### Purification of scFv

scFvs cloned in plasmid pET23NN were purified from the cytoplasm of BL21(DE3)pLysS and purified on a Ni-NTA column as described for the parental scFv13R4 [26].

### Cell transfection and immunofluorescence

HeLa cells were maintained in Dulbecco's modified Eagle's tissue culture medium (DMEM; Invitrogen) supplemented with L-glutamine (2 mM), penicillin (100 IU/ml), streptomycin (25 μg/ml) and 10% heat-inactivated fetal calf serum at 37°C in a humidified 5% CO_2 _atmosphere. Transient transfection was carried out with the TransFectin lipid reagent (Bio-Rad, Hercules, CA, USA) according to the manufacturer's instructions. Cells were seeded on coverslips in 6-well plates at 2.5 × 10^5 ^cells/well the day before transfection. 1 μg DNA and 2 μl of reagent diluted in 100 μl of DMEM were mixed and left at room temperature for 20 min. Cells were grown at 37°C for 24 h after addition of the mixture. The expressed GFP-tagged proteins were visualized after fixation of the transfected cells with 4% paraformaldehyde in PBS during 45 min at room temperature. After extensive wash with PBS, cells were dried and mounted with Fluoromount-G (SouthernBiotech, Birmingham, UK). The processed cells were examined with a Zeiss Axioplan fluorescence microscope equipped with an Olympus DP50 camera. Images were collected with a Zeiss 40× plan-neofluar objective and processed using Adobe Photoshop 5.5. For figure 8, HeLa were transfected with anti-histones clone 5 fused to the dsRed-monomer GFP, fixed as above and permeabilized with Triton ×-100 (0.2%, 5 min). The microtubule network was revealed with the 2F12C scFv (Table 2) at 3 μg/ml using the 9E10 anti-myc and an Alexa Fluor 488 anti-mouse IgG antibody. Cells were observed by confocal microscopy (×63).

## List of abbreviations

CAM : chloramphenicol;

CAT : chloramphenicol acethyl transferase;

CDR3 : complementary determining regions 3;

GFP : Green Fluorescent Protein;

H3 : heavy chain CDR3;

IF : Immunofluorescence;

K3 : κ light chain CDR3;

L3 : λ light chain CDR3;

Intrabodies, Intracellular antibodies; 

scFv : Single-chain Fv fragment.

## Authors' contributions

PP and AS performed the library construction and the selections. WW designed the spiked oligonucleotides. APS and JC performed the selection against E6 and the characterization of the anti-histones in HeLa. NB and CL participated in the characterization of the anti-tubulin. JGS supervised the oligonucleotide design and participated in the manuscript preparation. EW and PM designed the approach, supervised the work and wrote the manuscript. All authors read and approved the final manuscript.

## Supplementary Material

Additional File 1 **Amino acid distribution in CDR3s**. Plot of the amino acid distribution in the collected database of human CDR3 sequences (Yellow bars) and encoded by the spiked oligonucleotides (Red bars). The values for the oligonucleotides are predicted from the degeneracy (see additional file 2: Sequences of the spiked oligonucleotides used to introduce the random CDR3 loops). Stop codons are noted *. H3_n_p is the p^th ^amino acid of the n amino acid long VH CDR3 loops. K3_n_p and L3_n_p are the frequencies for the light chain CDR3 from, respectively, κ and λ classes. Click here for file

Additional File 2 **Sequences of the spiked oligonucleotides used to introduce the random CDR3 loops**. H3_n = n amino acid long VH CDR3 loop; K3_n = n amino acid long VL κ CDR3 loop; L3_n = n amino acid long VL λ CDR3 loop. For the degenerated positions, the percentages of the 4 bases are given as N(A/C/G/T) Click here for file

Additional File 3 **Sequence of randomly picked clones**. Sequences of 118 scFvs randomly picked in the library. For some clones the sequence quality was not good enough to read the light chain CDR3 and are noted nr (non-read). Click here for file

Additional File 4 ***In vitro *characterization of some anti-histones scFvs**. In cell screened anti-histones (Figure 6) were expressed and purified from *E. coli*. (A) sequence of the clones. (B) reactivity measured by western blot and (C) dot blot. The histones preparation used for the selection was from Sigma. "histones Roche" is another histones preparation obtained from Roche. MBP: Maltose Binding Protein. T: tubulin clone 2F12C (Table 2). C: negative control (no scFv). The sequences of the clones 2, 5, 6, 9 and 10 have been deposited in the EMBL database and their accession numbers are respectively AM888346AM888346, AM888347, AM888348, AM888349 and AM888350AM888350. Click here for file

Additional File 5 **Biacore analysis of clone 2F12C**. ScFv affinity was determined on a BIACORE 2000 apparatus (Biacore AB, Uppsala, Sweden). HBS-EP is 0.01 M HEPES, pH 7.4, 0.15 M NaCl, 3 mM EDTA, 0.005% P20 surfactant. Anti-myc antibody (purified 9E10 from Sigma) was covalently immobilized on a flow cell of a carboxymethyl dextran sensorchip (CM5 from Biacore AB) using the amine coupling method according to the manufacturer's instructions. The immobilization level was around 4000 resonance units (RU). A second flowcell was treated with the same chemical procedure without the 9E10 and used as a reference. After the injection of the scFv (50 μg/ml in HBS-EP containing 0.1% dextran), different concentrations of tubulin in HBS-EP were injected during 180 s over the two flowcells and followed by a dissociation step of 400s. The experiments were performed at a 50 μl/min flow rate at 25°C. Between each run, sensor surfaces were regenerated with a pulse of 25 mM HCl. All the sensorgrams were corrected by subtracting the signal from the reference flowcell and were globally fitted using BIAevaluation version 3.2 software (Biacore AB) to a two-state reaction model (A+B←kd1→ka1AB←kd2→ka2AB∗
 MathType@MTEF@5@5@+=feaafiart1ev1aaatCvAUfKttLearuWrP9MDH5MBPbIqV92AaeXatLxBI9gBaebbnrfifHhDYfgasaacPC6xNi=xH8viVGI8Gi=hEeeu0xXdbba9frFj0xb9qqpG0dXdb9aspeI8k8fiI+fsY=rqGqVepae9pg0db9vqaiVgFr0xfr=xfr=xc9adbaqaaeGacaGaaiaabeqaaeqabiWaaaGcbaGaemyqaeKaey4kaSIaemOqai0aa0baaSqaamaaoGbameqabaGaem4AaSMaemizaqMaeGymaedaliaawcziaaqaamaaoqcameaacqWGRbWAcqWGHbqycqaIXaqmaeqaliaawkziaaaakiabdgeabjabdkeacnaaDaaaleaadaGdyaadbeqaaiabdUgaRjabdsgaKjabikdaYaWccaGLqgcaaeaadaGdKaadbaGaem4AaSMaemyyaeMaeGOmaidabeWccaGLsgcaaaGccqWGbbqqcqWGcbGqdaahaaWcbeqaaiabgEHiQaaaaaa@494B@), where ka1 and kd1 are the association and dissociation rate constants for the first equilibrium, and ka2 and kd2 for the second. ka1 = (4.39 ± 0.06) 10^4 ^M^-1 ^s^-1^; kd1 = (3,65 ± 0,10) 10^-2 ^s^-1^; ka2 = (1.37 ± 0.0178) 10^-2 ^s^-1^; kd2 = (8.03 ± 0.113) 10^-4 ^s^-1^; Kd = (kd1 × kd2)/(ka1 × ka2) = 49 ± 3 nM (χ^2 ^= 0,732). Click here for file
