# Supplementary material for: A focused antibody library for selecting scFvs expressed at high levels in the cytoplasm
Source: BMC Biotechnol. 2007 Nov 22;7:81. doi: 10.1186/1472-6750-7-81 (PMC2241821; doi:10.1186/1472-6750-7-81)
Supplement: Additional File 1 — Amino acid distribution in CDR3s. Plot of the amino acid distribution in the collected database of human CDR3 sequences (Yellow bars) and encoded by the spiked oligonucleotides (Red bars). The values for the oligonucleotides are predicted from the degeneracy (see additional file 2: Sequences of the spiked oligonucleotides used to introduce the random CDR3 loops). Stop codons are noted *. H3_n_p is the pth amino acid of the n amino acid long VH CDR3 loops. K3_n_p and L3_n_p are the frequencies for the light chain CDR3 from, respectively, κ and λ classes. [file 1472-6750-7-81-S1.pdf]

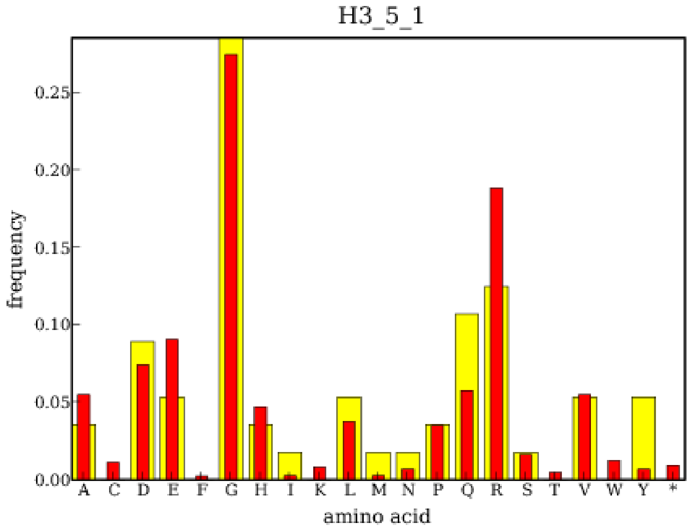
H3_5_1
frequency
amino acid

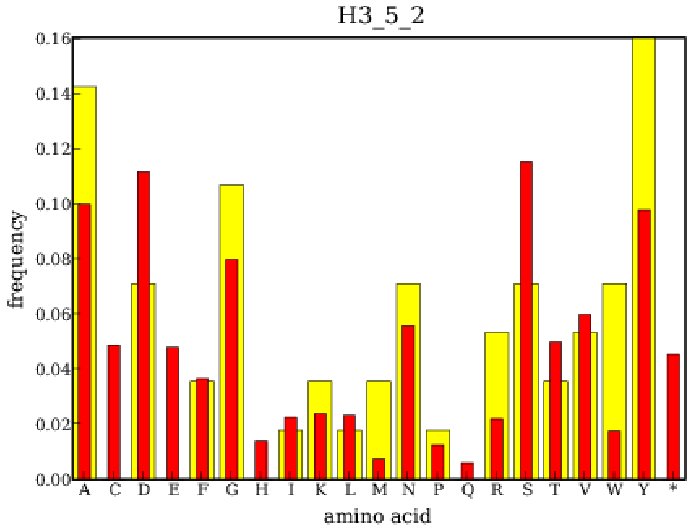
H3_5_2
frequency
amino acid

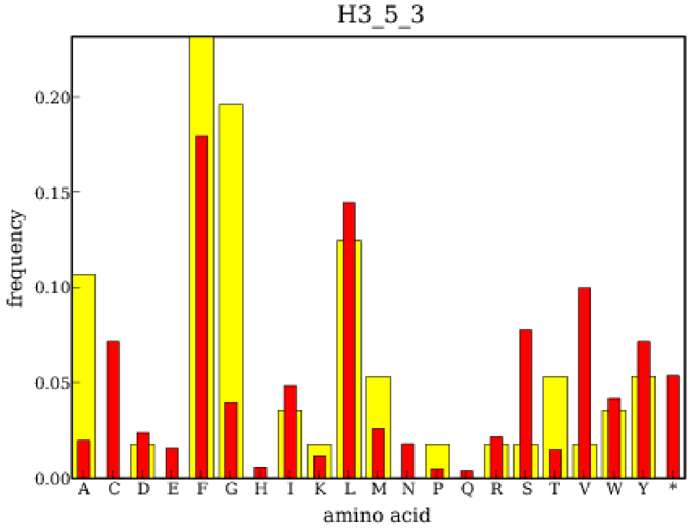
H3_5_3
frequency
amino acid

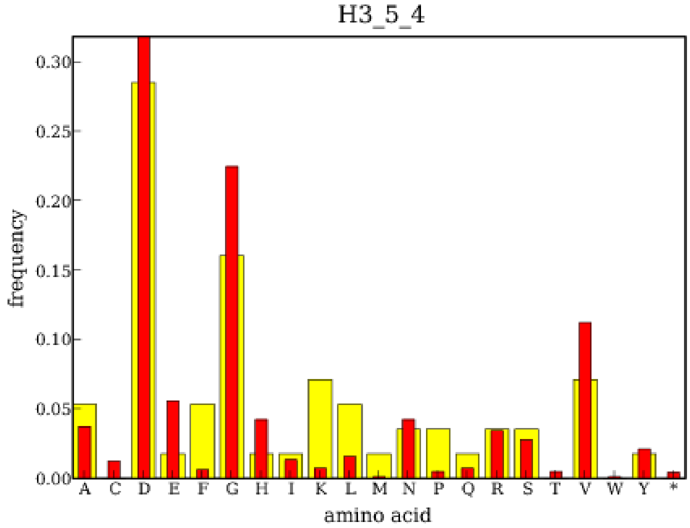
H3_5_4
frequency
amino acid

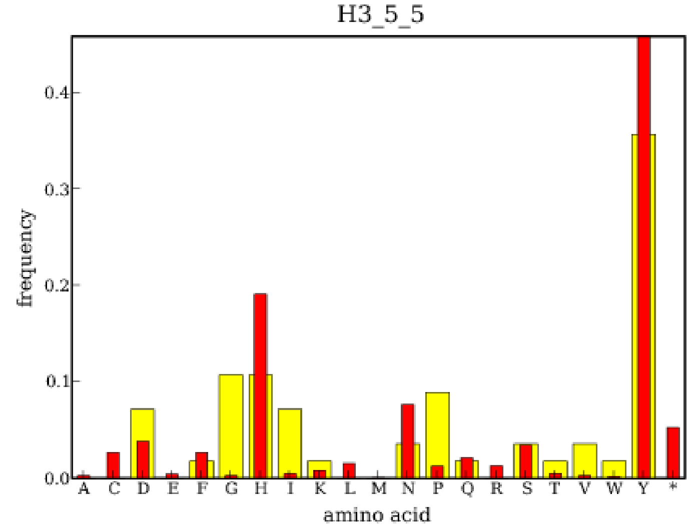
H3_5_5
frequency
amino acid

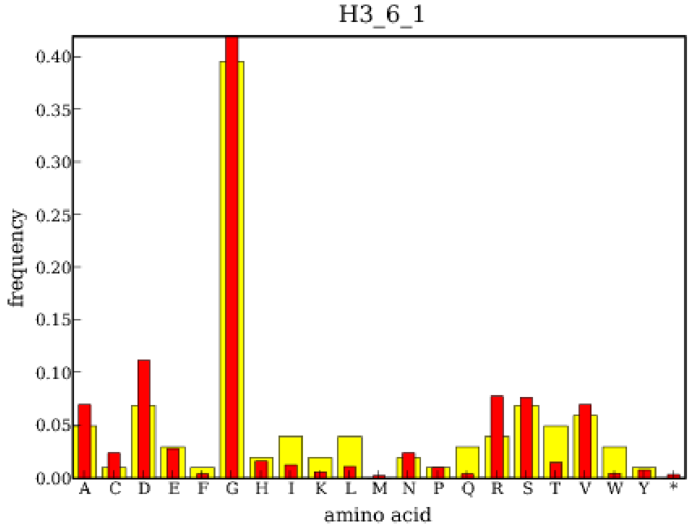
H3_6_1
frequency
amino acid

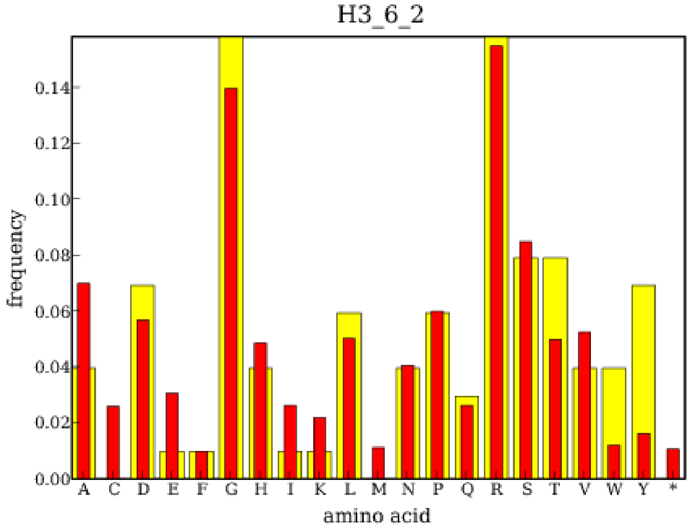
H3_6_2
frequency
amino acid

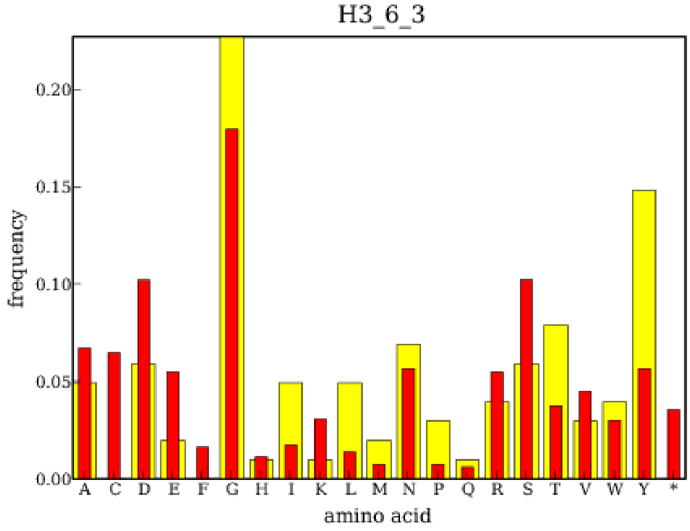
H3_6_3
frequency
amino acid

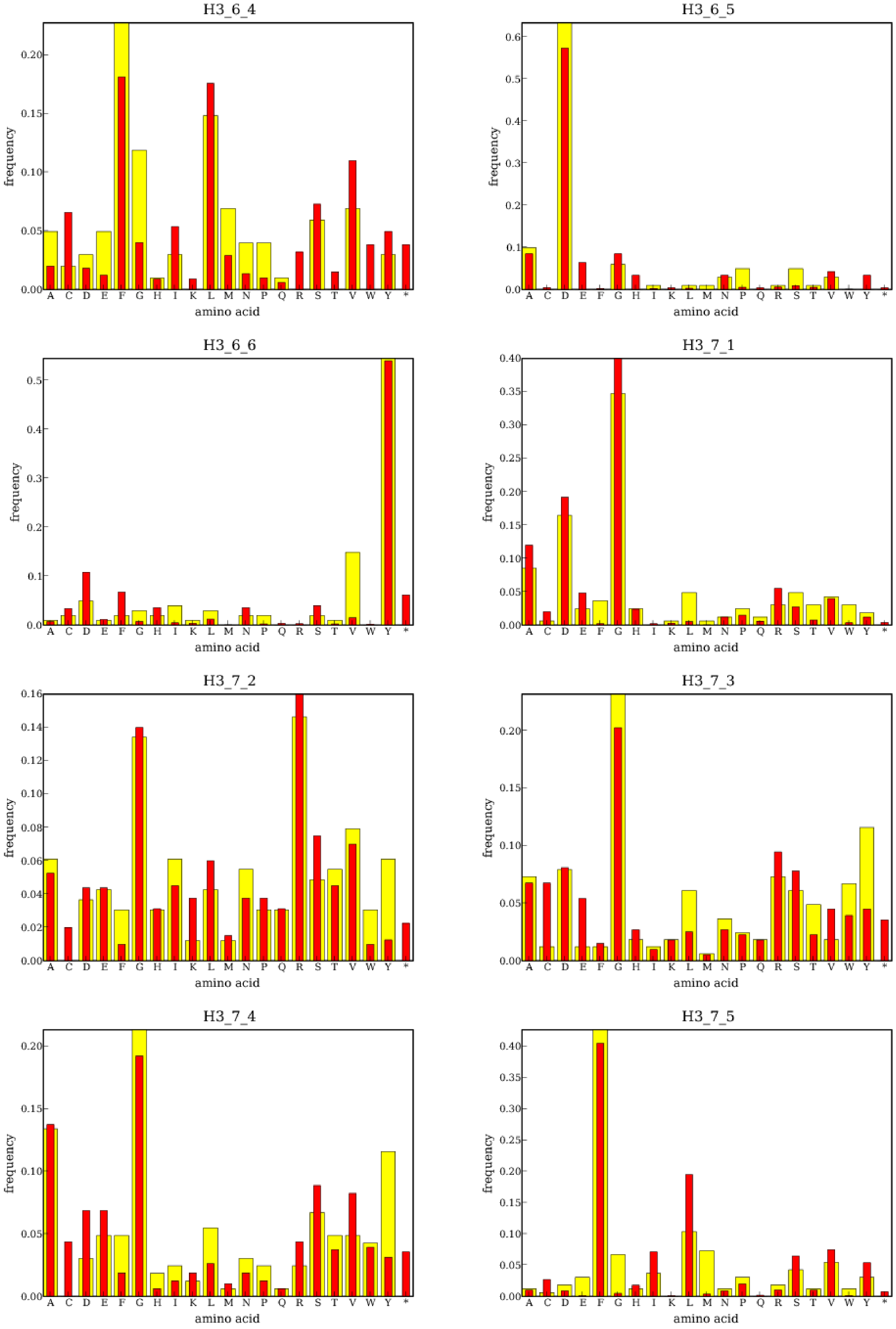
H3_6_4
H3_6_5
H3_6_6
H3_7_1
H3_7_2
H3_7_3
H3_7_4
H3_7_5
frequency
amino acid
A C D E F G H I K L M N P Q R S T V W Y *

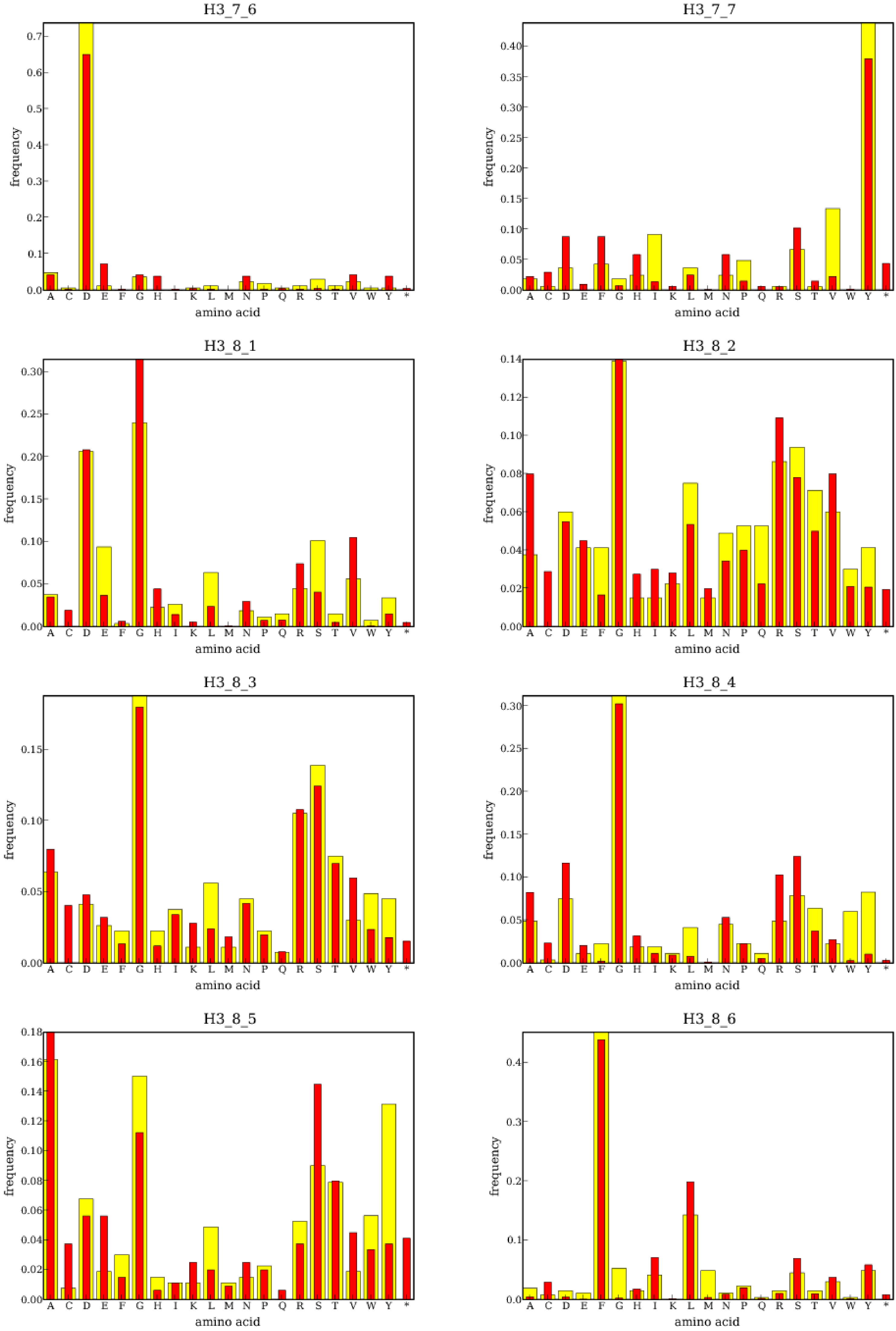
H3_7_6
H3_7_7
H3_8_1
H3_8_2
H3_8_3
H3_8_4
H3_8_5
H3_8_6
frequency
amino acid
A C D E F G H I K L M N P Q R S T V W Y *

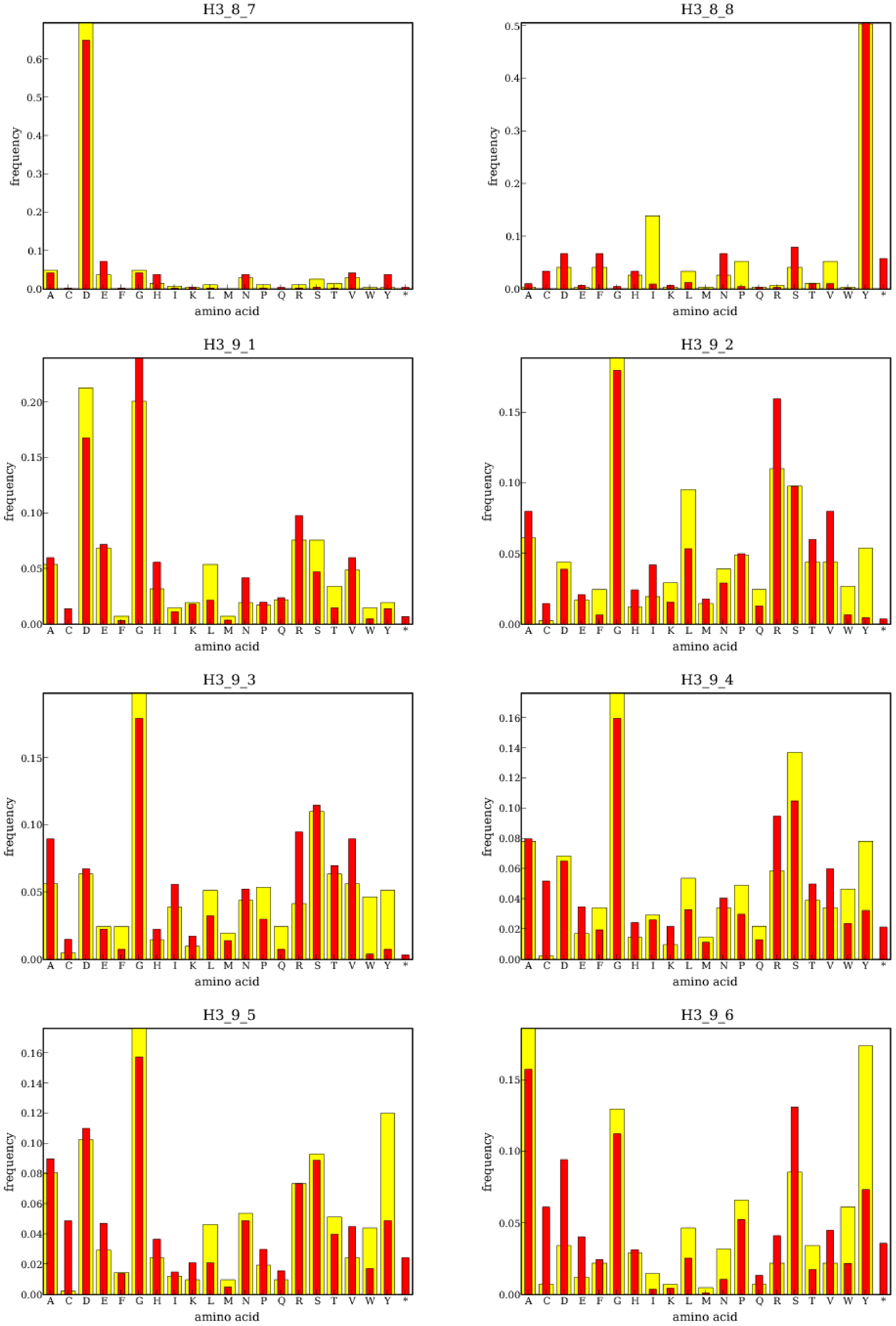
H3_8_7
H3_8_8
H3_9_1
H3_9_2
H3_9_3
H3_9_4
H3_9_5
H3_9_6
frequency
amino acid
A C D E F G H I K L M N P Q R S T V W Y *

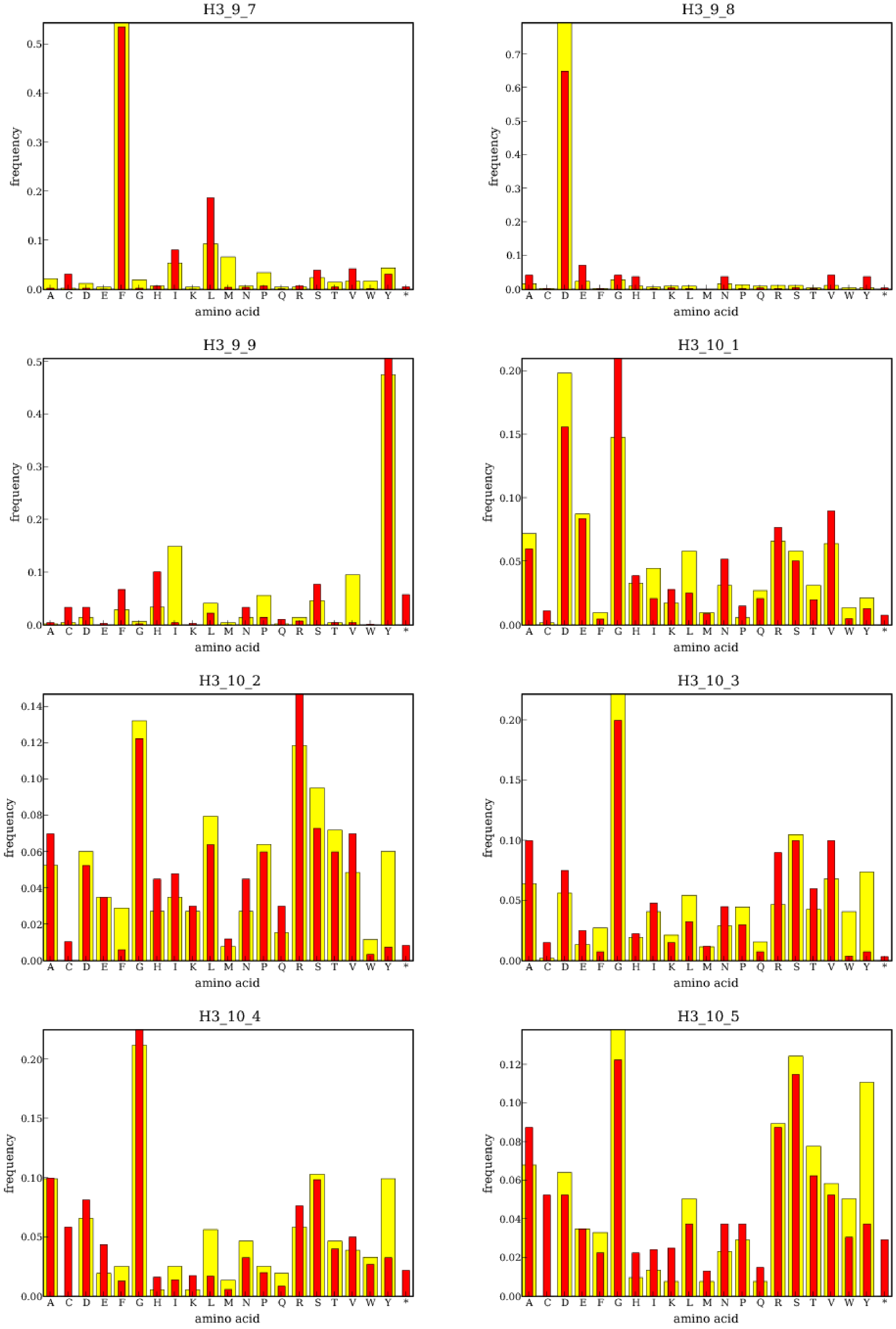
H3_9_7
H3_9_8
H3_9_9
H3_10_1
H3_10_2
H3_10_3
H3_10_4
H3_10_5
frequency
amino acid
A C D E F G H I K L M N P Q R S T V W Y *

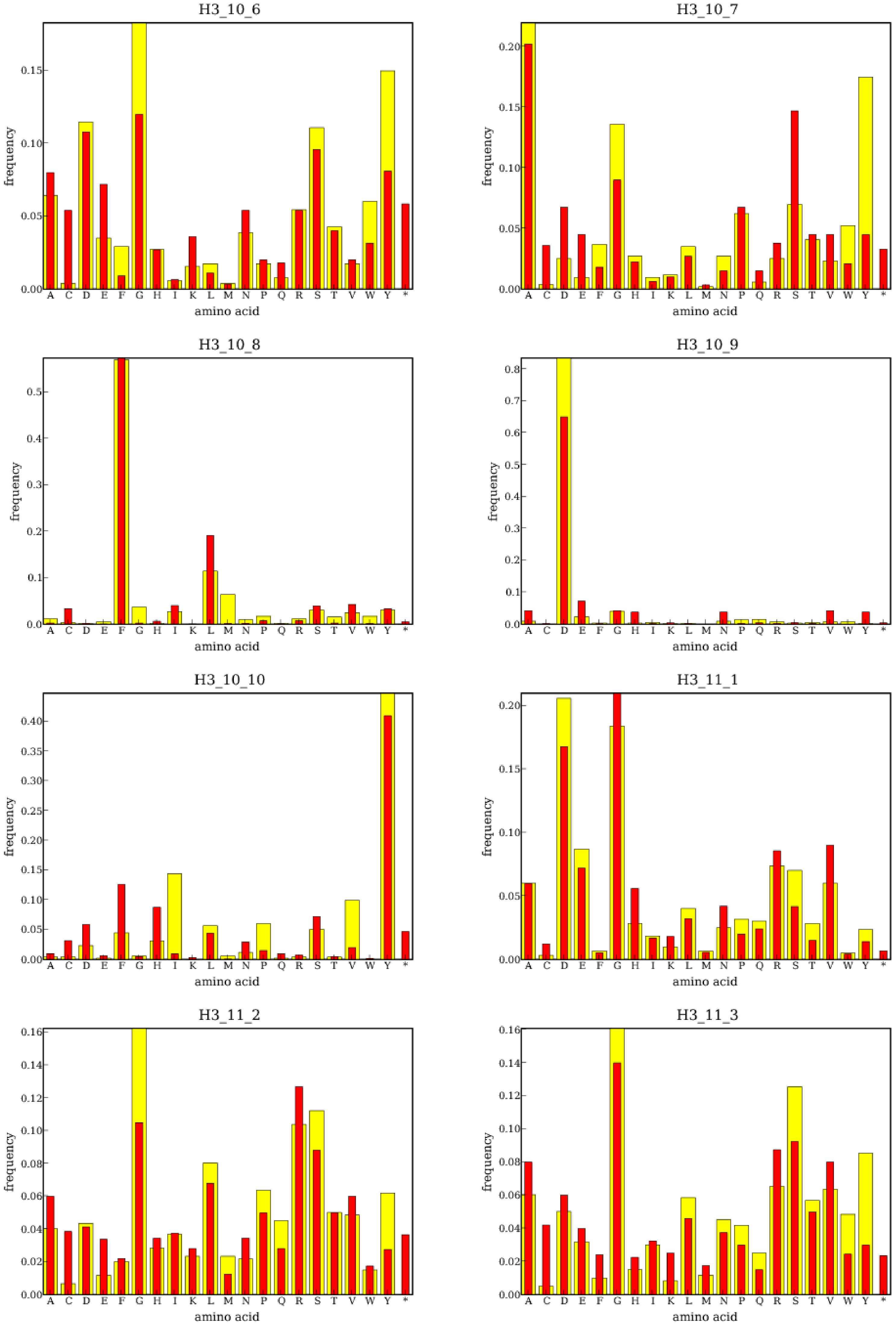
H3_10_6
H3_10_7
H3_10_8
H3_10_9
H3_10_10
H3_11_1
H3_11_2
H3_11_3
frequency
amino acid
A C D E F G H I K L M N P Q R S T V W Y *

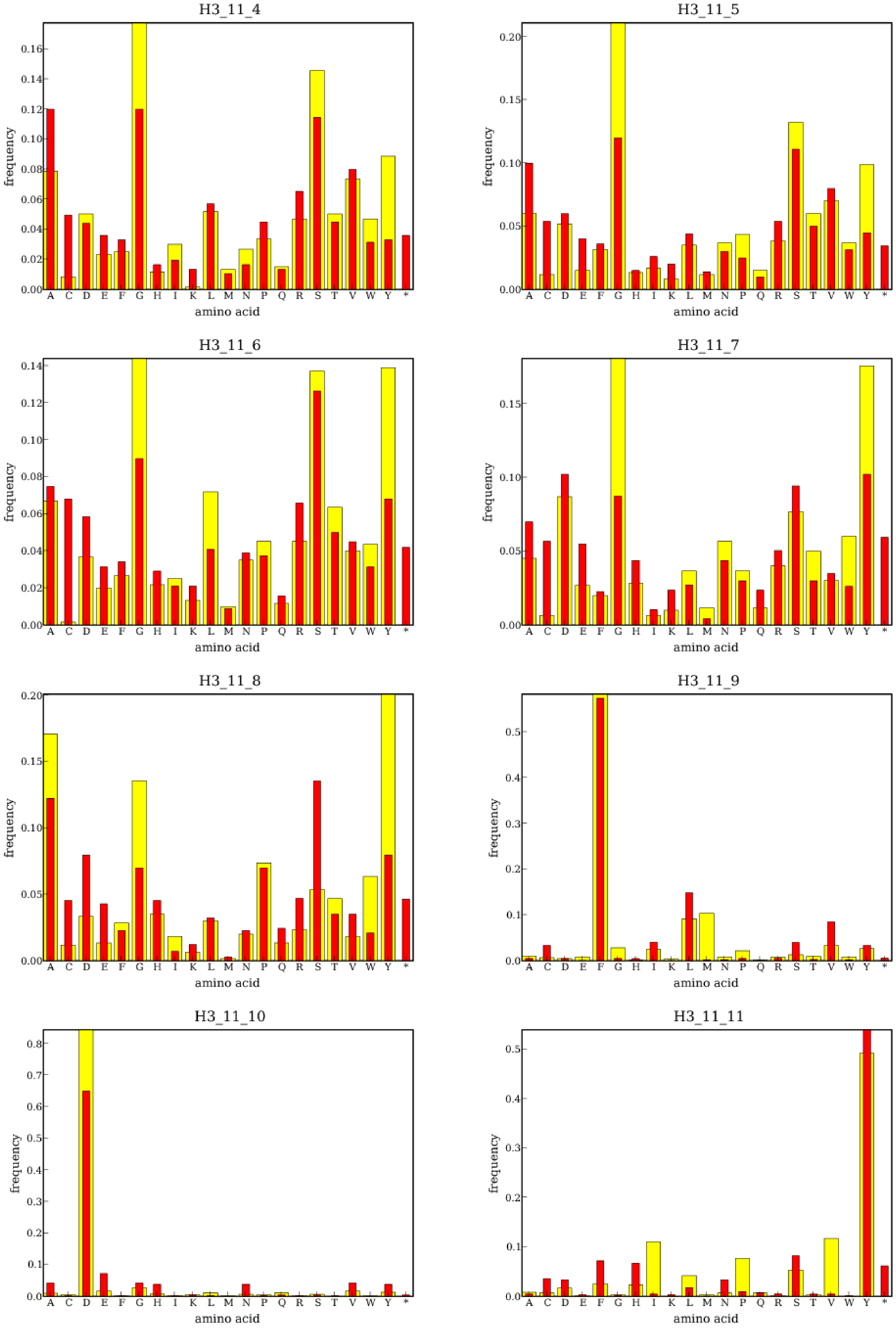
H3_11_4
H3_11_5
H3_11_6
H3_11_7
H3_11_8
H3_11_9
H3_11_10
H3_11_11
frequency
amino acid
A C D E F G H I K L M N P Q R S T V W Y *

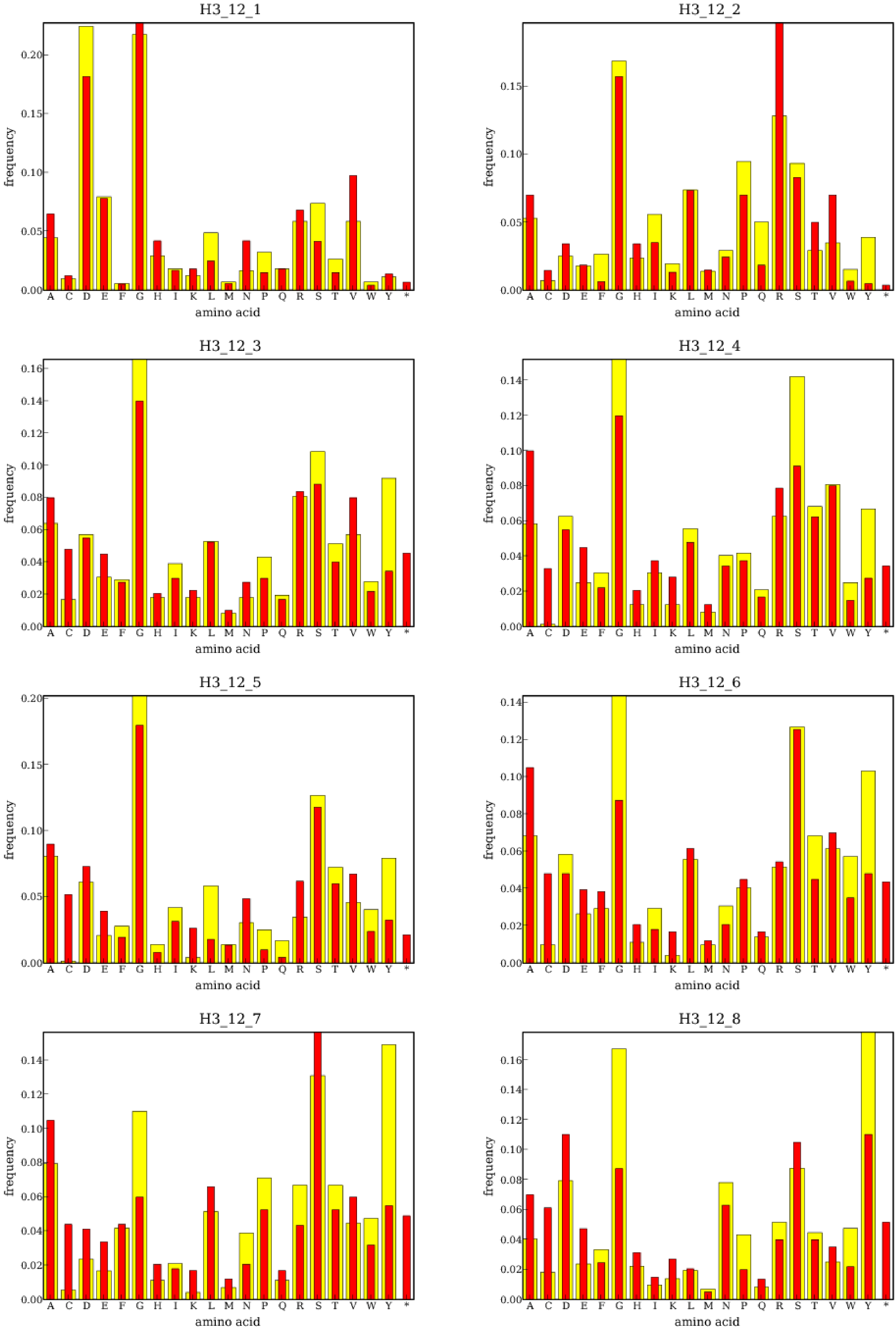
H3_12_1
H3_12_2
H3_12_3
H3_12_4
H3_12_5
H3_12_6
H3_12_7
H3_12_8
frequency
amino acid
A C D E F G H I K L M N P Q R S T V W Y *

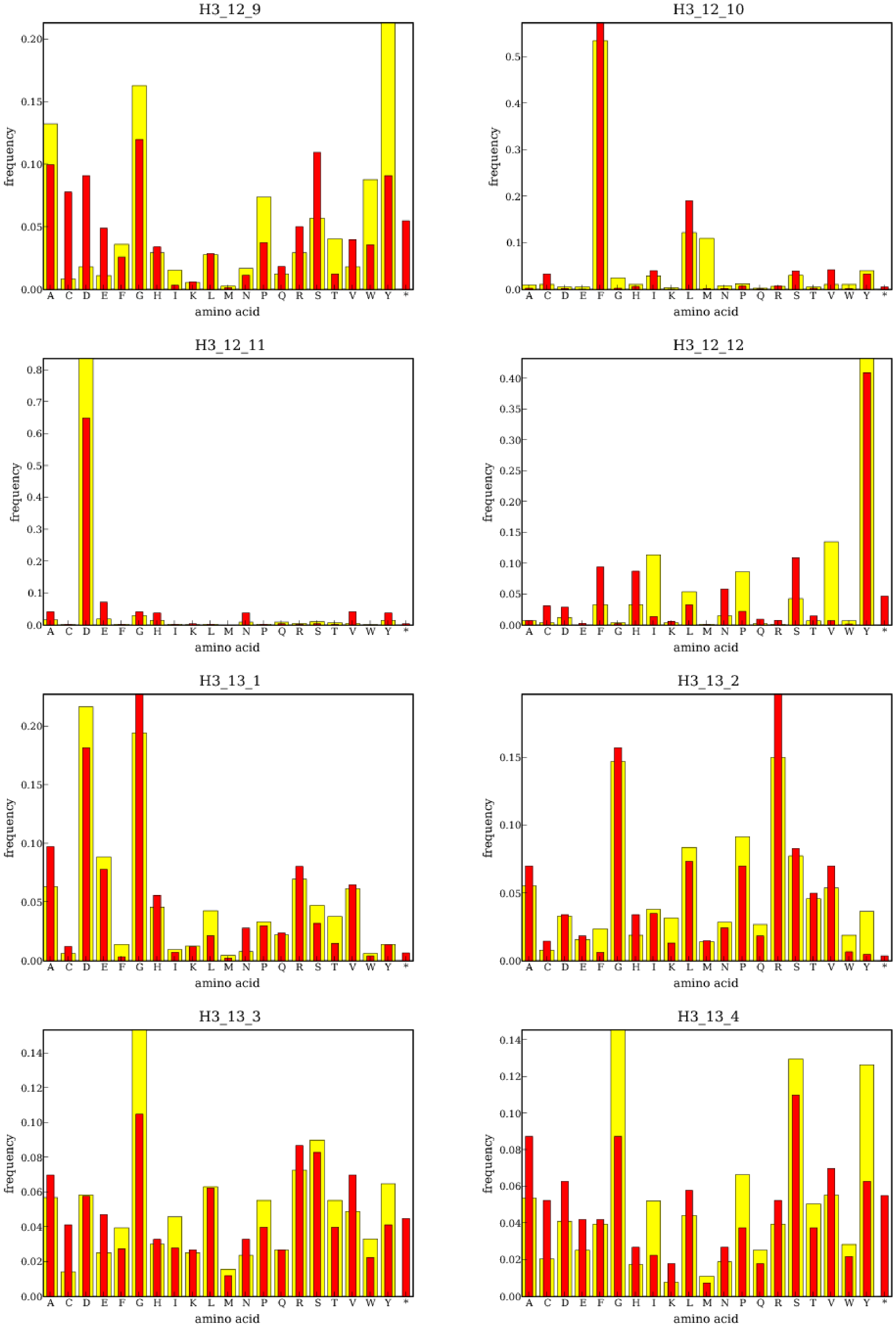
H3_12_9
H3_12_10
H3_12_11
H3_12_12
H3_13_1
H3_13_2
H3_13_3
H3_13_4
frequency
amino acid
A C D E F G H I K L M N P Q R S T V W Y *

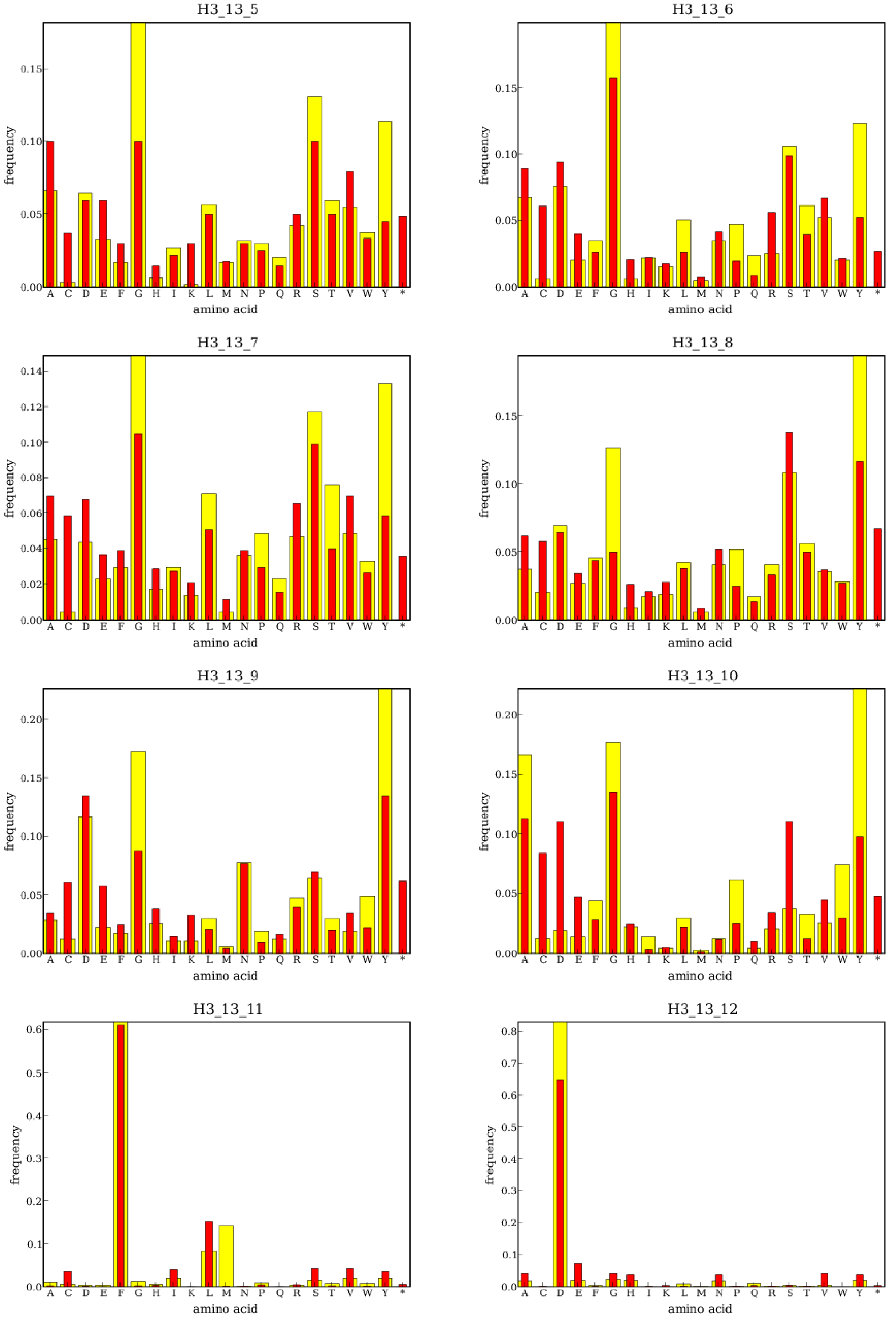
H3_13_5
H3_13_6
H3_13_7
H3_13_8
H3_13_9
H3_13_10
H3_13_11
H3_13_12
frequency
amino acid
A C D E F G H I K L M N P Q R S T V W Y *

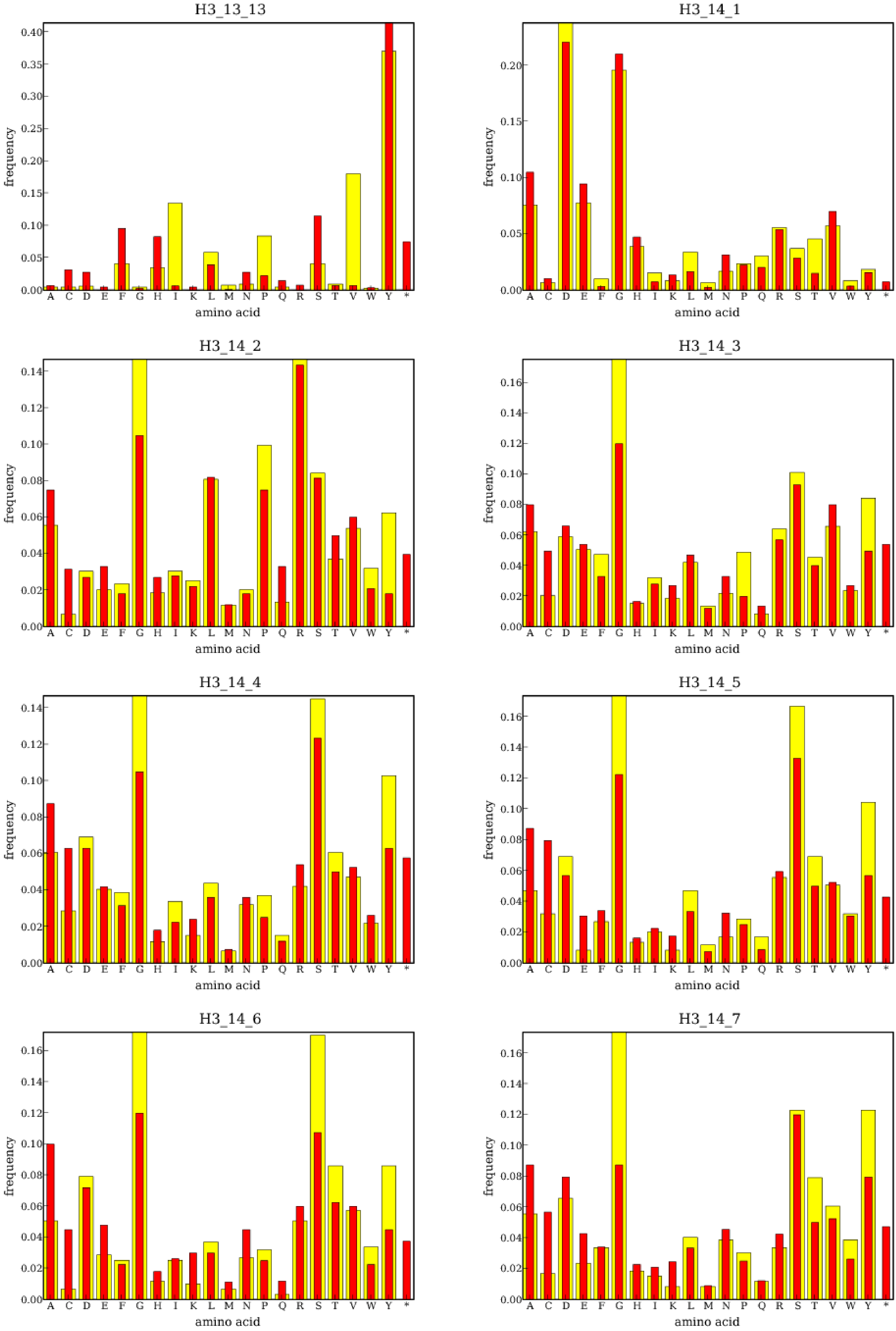
H3_13_13
H3_14_1
H3_14_2
H3_14_3
H3_14_4
H3_14_5
H3_14_6
H3_14_7
frequency
amino acid
A C D E F G H I K L M N P Q R S T V W Y *

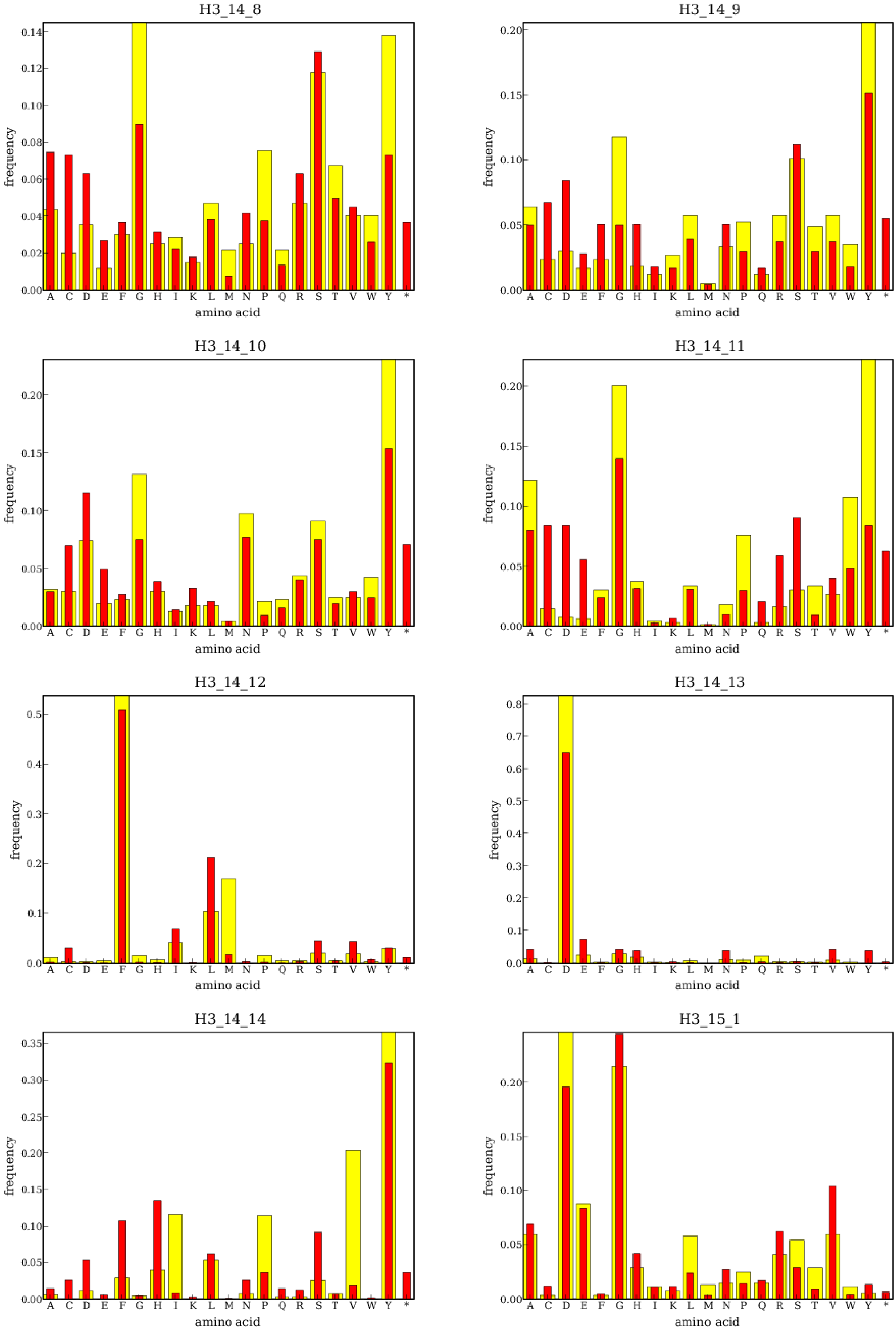
H3_14_8
H3_14_9
H3_14_10
H3_14_11
H3_14_12
H3_14_13
H3_14_14
H3_15_1
frequency
amino acid
A C D E F G H I K L M N P Q R S T V W Y *

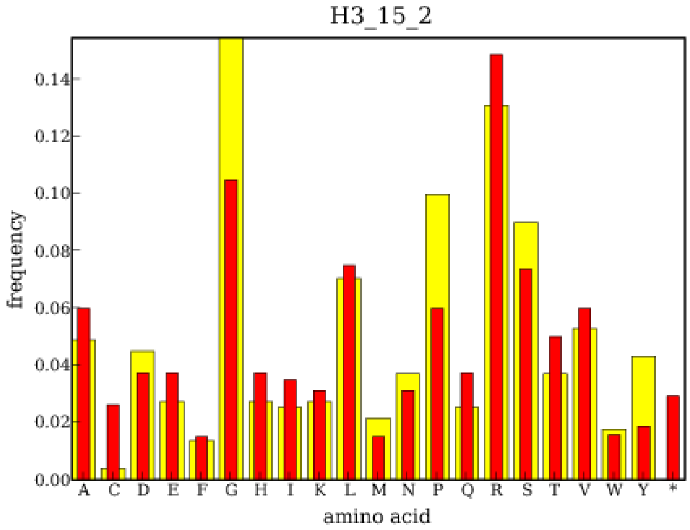
H3_15_2
frequency
amino acid
A C D E F G H I K L M N P Q R S T V W Y *

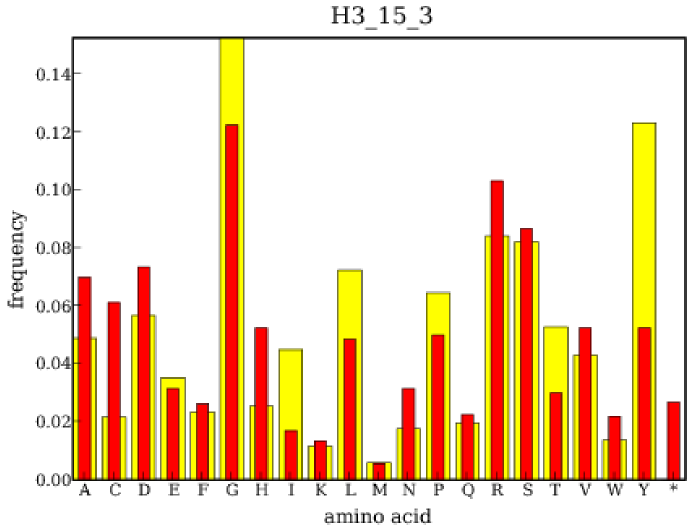
H3_15_3
frequency
amino acid
A C D E F G H I K L M N P Q R S T V W Y *

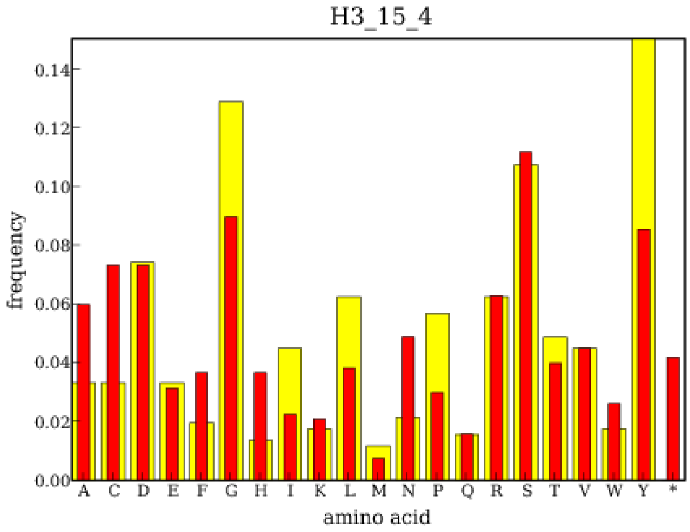
H3_15_4
frequency
amino acid
A C D E F G H I K L M N P Q R S T V W Y *

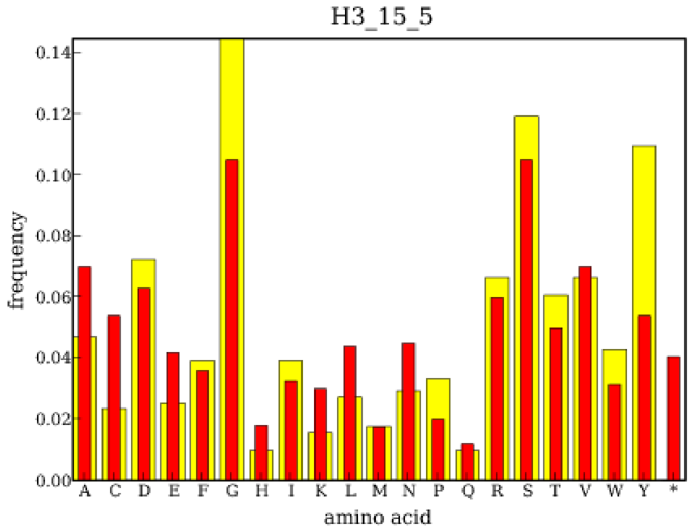
H3_15_5
frequency
amino acid
A C D E F G H I K L M N P Q R S T V W Y *

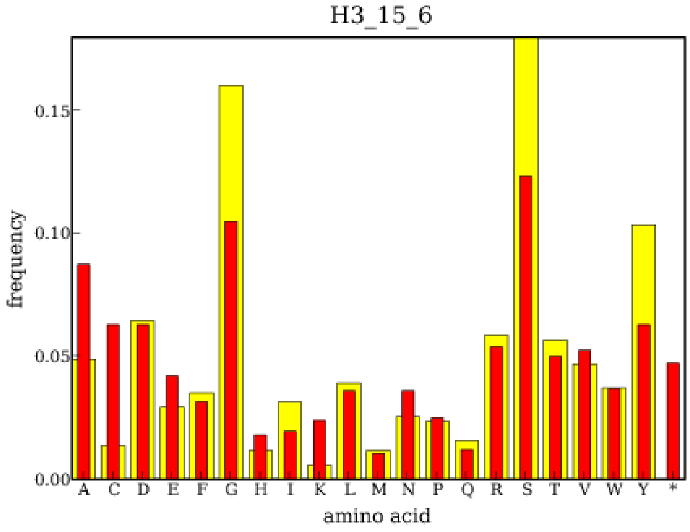
H3_15_6
frequency
amino acid
A C D E F G H I K L M N P Q R S T V W Y *

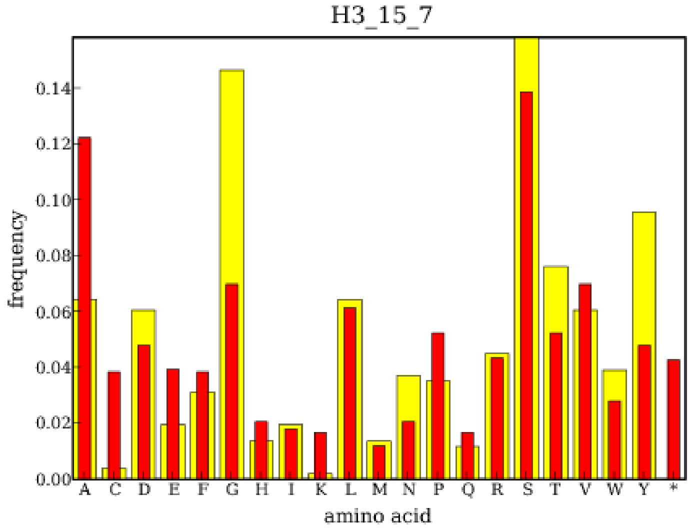
H3_15_7
frequency
amino acid
A C D E F G H I K L M N P Q R S T V W Y *

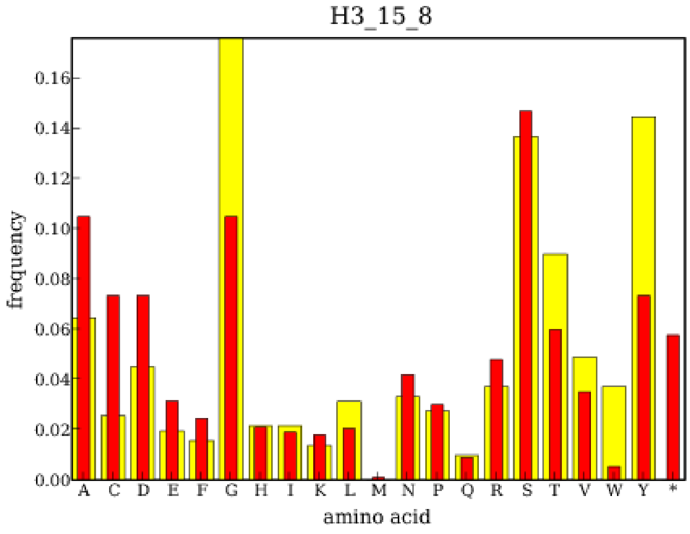
H3_15_8
frequency
amino acid
A C D E F G H I K L M N P Q R S T V W Y *

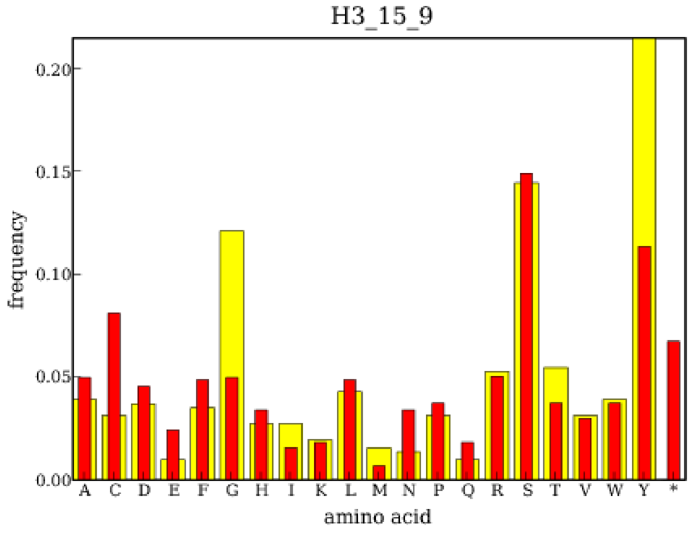
H3_15_9
frequency
amino acid
A C D E F G H I K L M N P Q R S T V W Y *

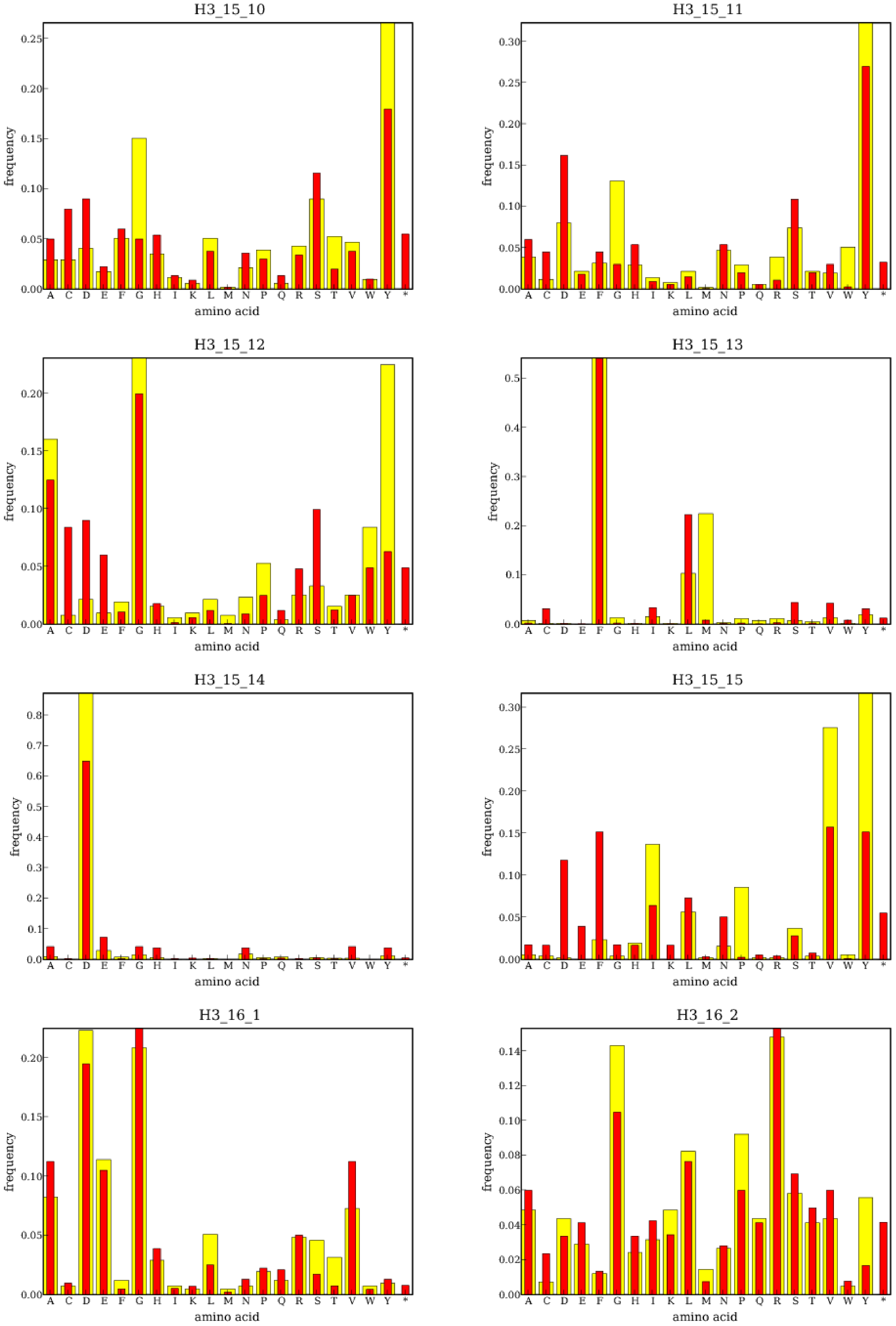
H3_15_10
H3_15_11
H3_15_12
H3_15_13
H3_15_14
H3_15_15
H3_16_1
H3_16_2
frequency
amino acid
A C D E F G H I K L M N P Q R S T V W Y *

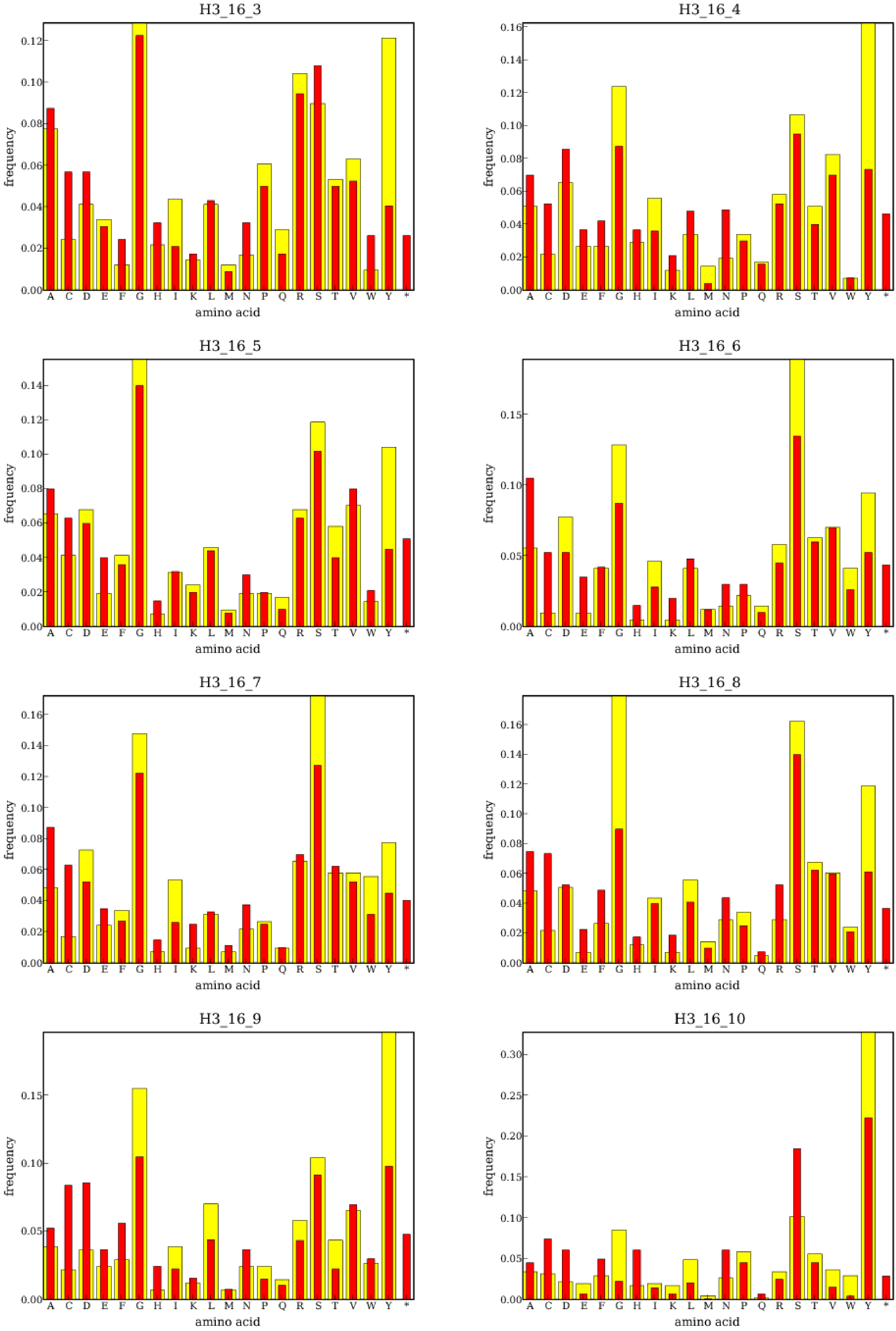
H3_16_3
H3_16_4
H3_16_5
H3_16_6
H3_16_7
H3_16_8
H3_16_9
H3_16_10
frequency
amino acid
A C D E F G H I K L M N P Q R S T V W Y *

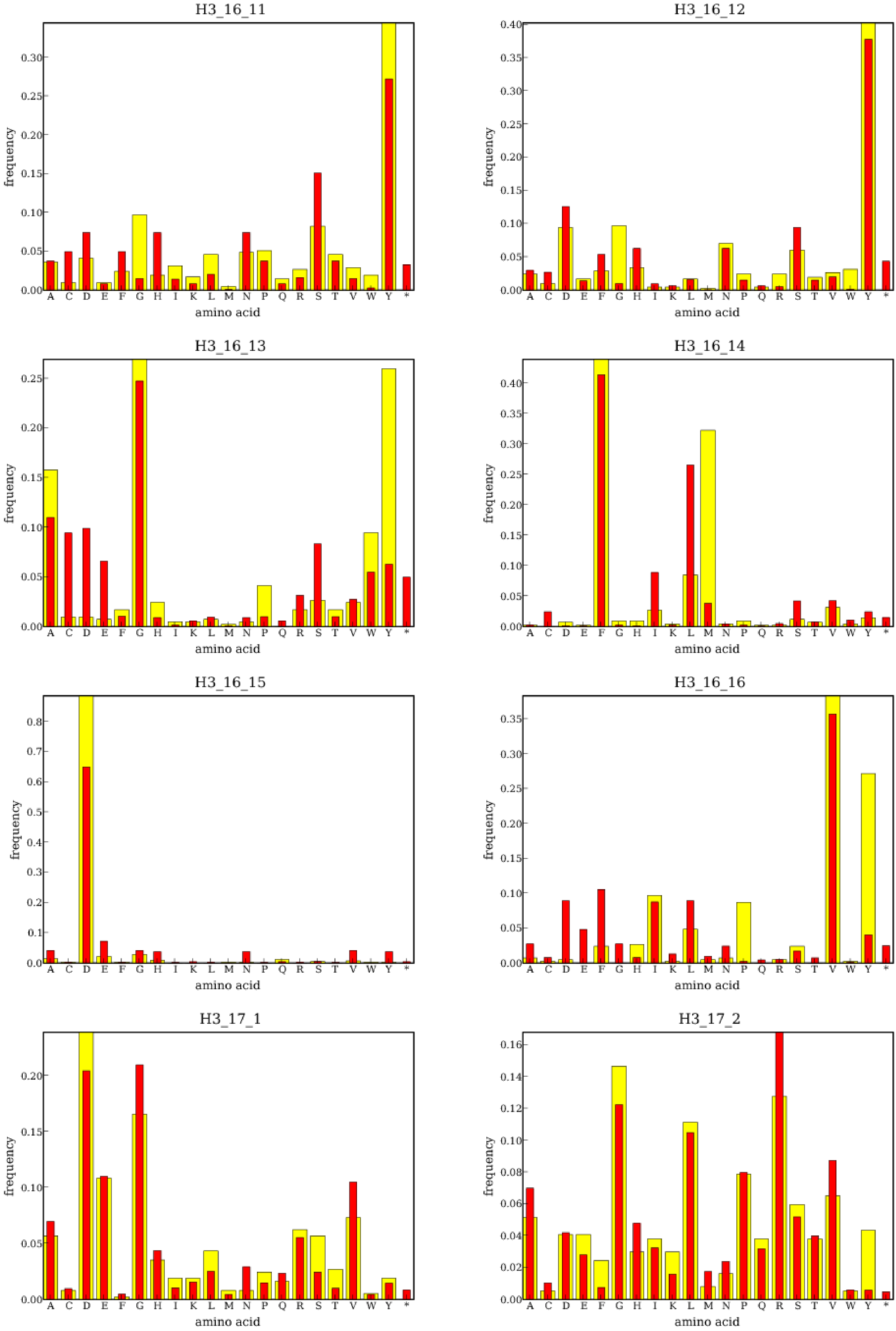
H3_16_11
H3_16_12
H3_16_13
H3_16_14
H3_16_15
H3_16_16
H3_17_1
H3_17_2
frequency
amino acid
A C D E F G H I K L M N P Q R S T V W Y *

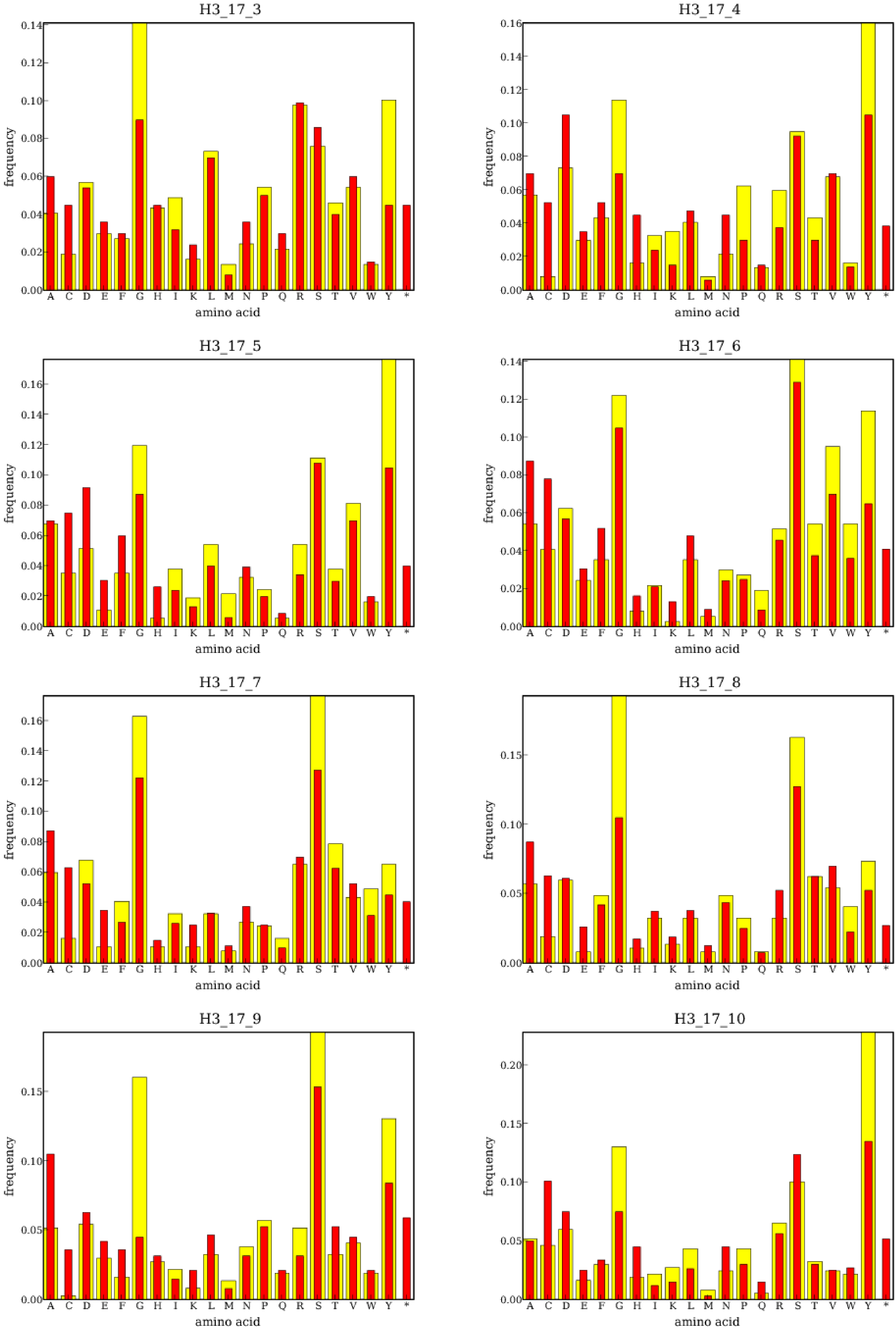
H3_17_3
H3_17_4
H3_17_5
H3_17_6
H3_17_7
H3_17_8
H3_17_9
H3_17_10
frequency
amino acid
A C D E F G H I K L M N P Q R S T V W Y *

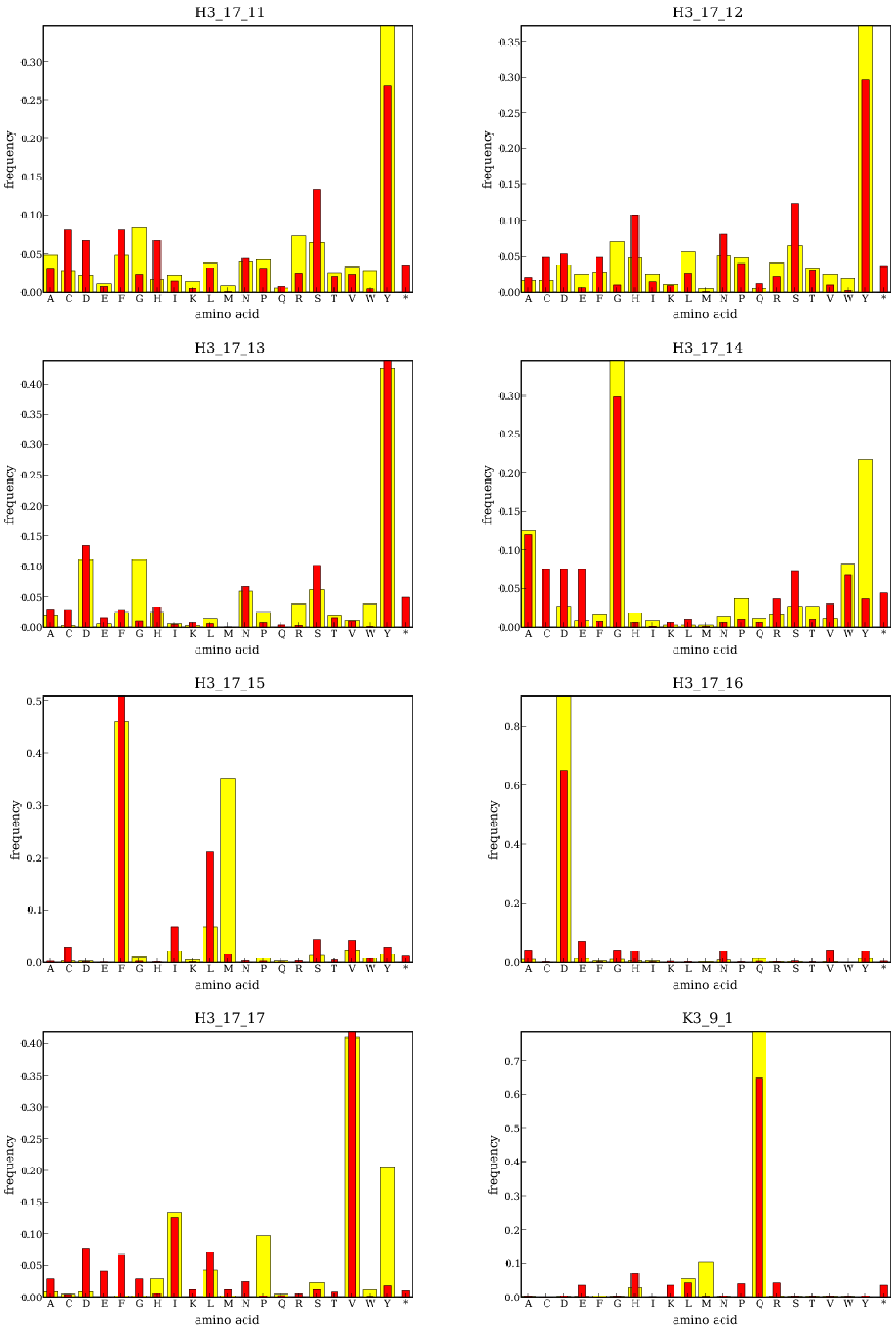
H3_17_11
H3_17_12
H3_17_13
H3_17_14
H3_17_15
H3_17_16
H3_17_17
K3_9_1
frequency
amino acid
A C D E F G H I K L M N P Q R S T V W Y *

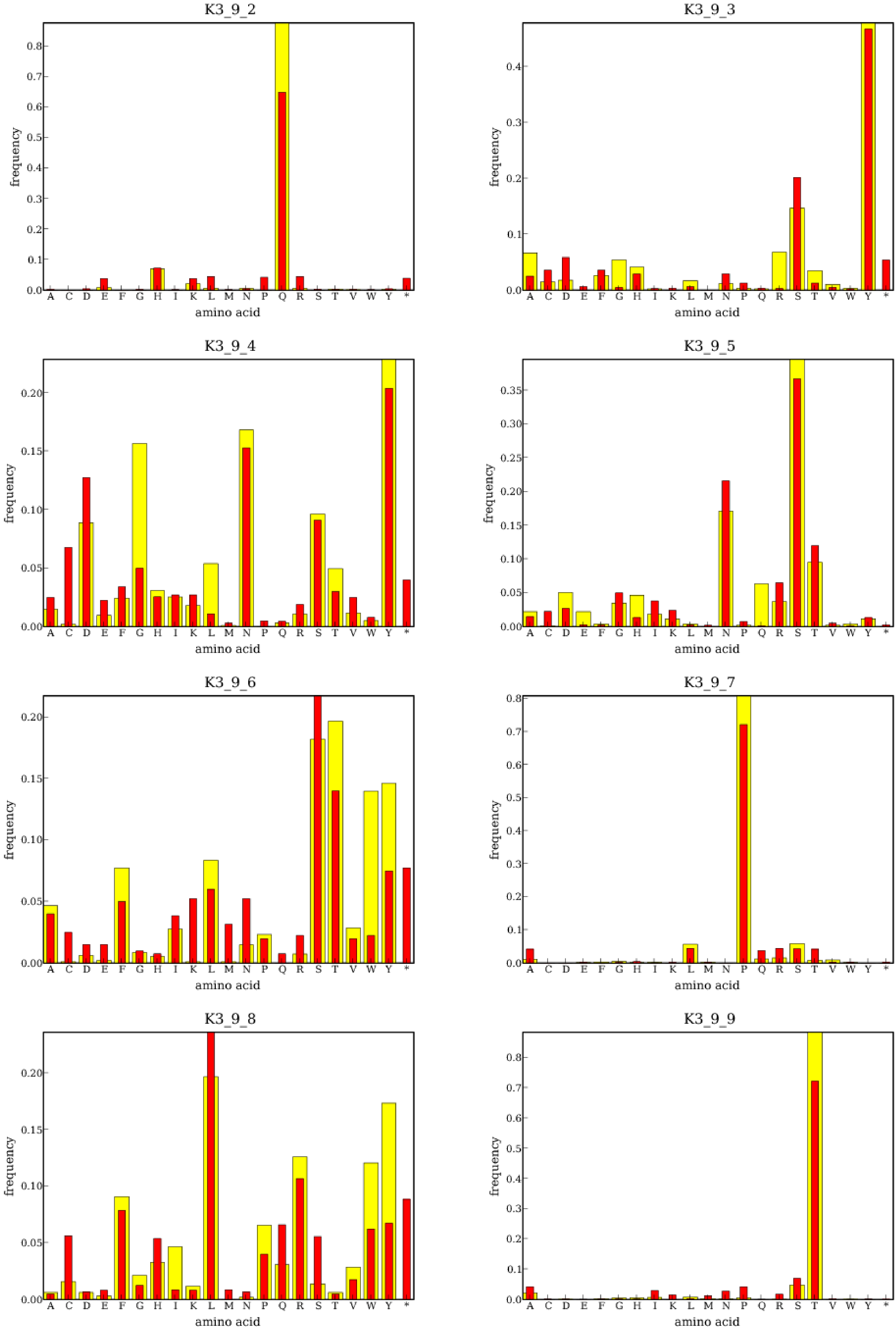
K3_9_2
K3_9_3
K3_9_4
K3_9_5
K3_9_6
K3_9_7
K3_9_8
K3_9_9
frequency
amino acid
A C D E F G H I K L M N P Q R S T V W Y *

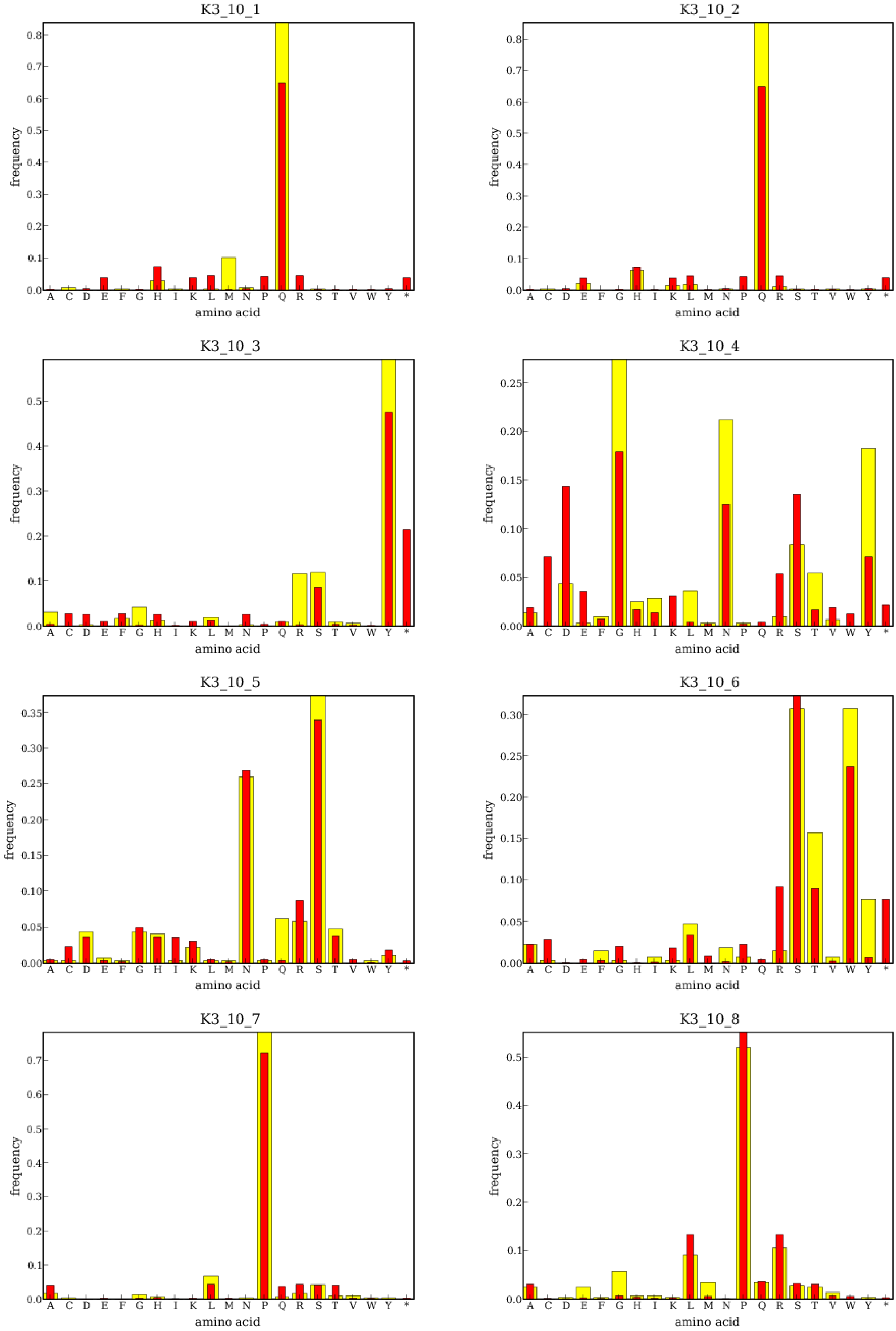
K3_10_1
K3_10_2
K3_10_3
K3_10_4
K3_10_5
K3_10_6
K3_10_7
K3_10_8
frequency
amino acid
A C D E F G H I K L M N P Q R S T V W Y *

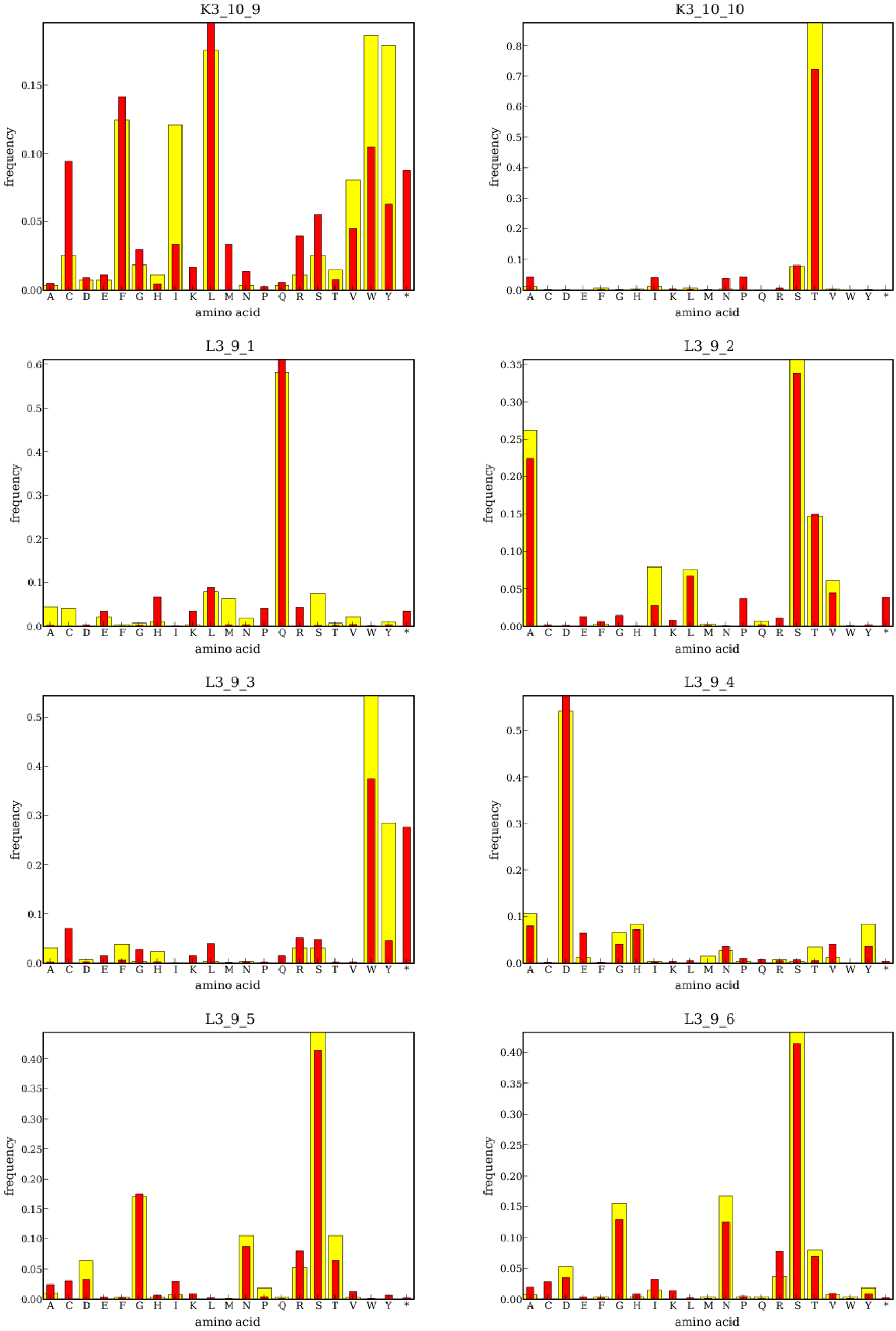
K3_10_9
K3_10_10
L3_9_1
L3_9_2
L3_9_3
L3_9_4
L3_9_5
L3_9_6
frequency
amino acid
A C D E F G H I K L M N P Q R S T V W Y *

L3_9_7

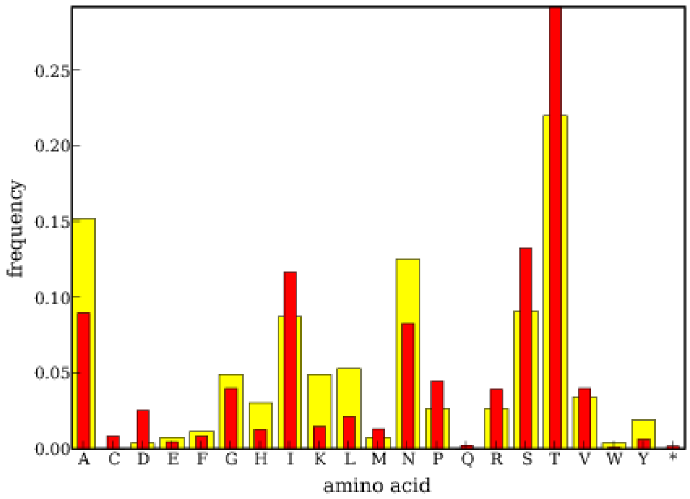

L3_9_8

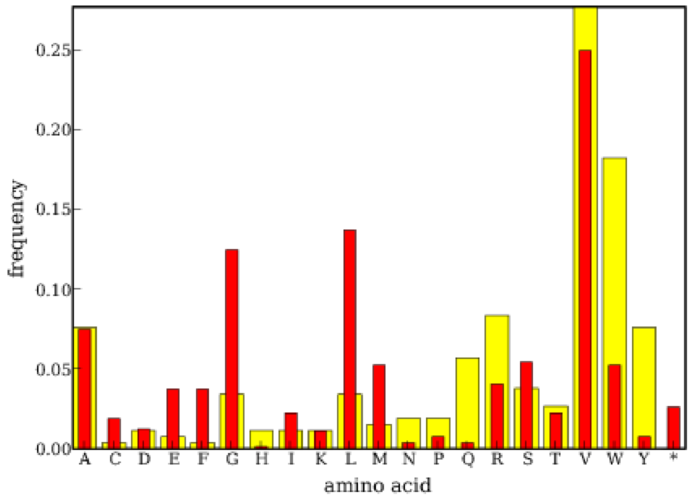

L3_9_9

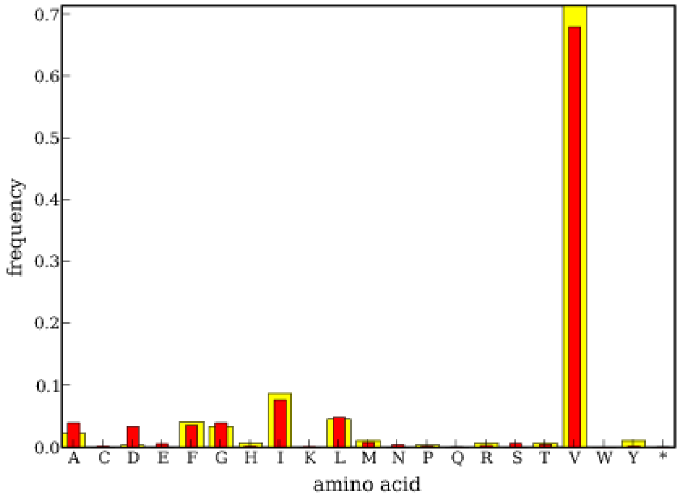

L3_10_1

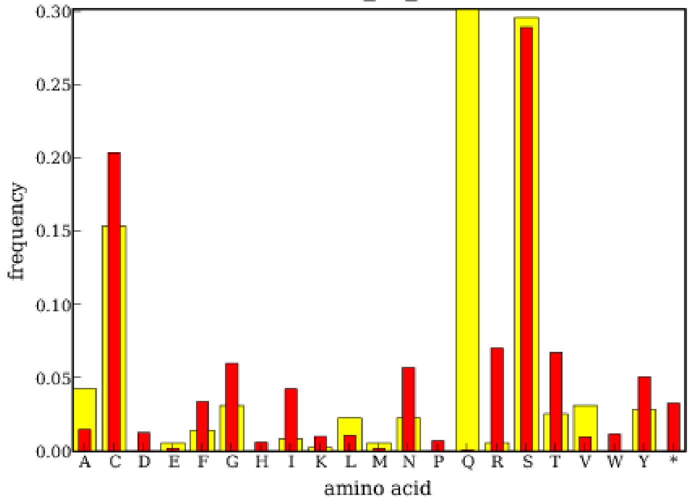

L3_10_2

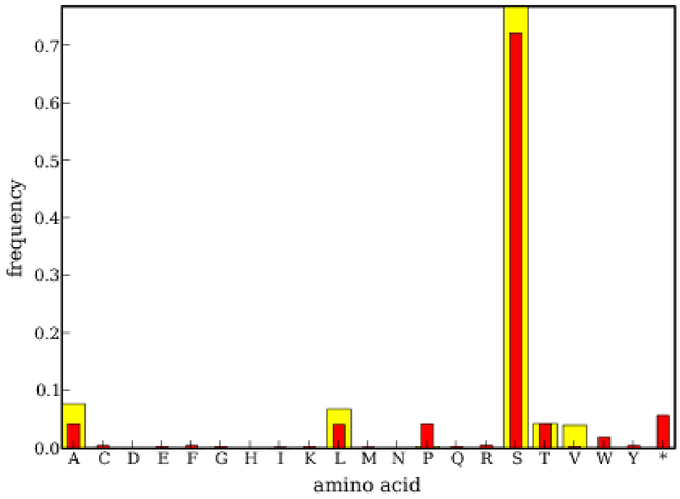

L3_10_3

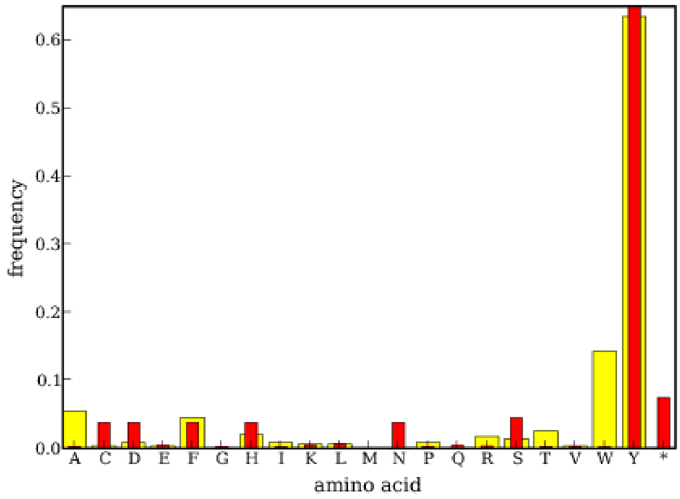

L3_10_4

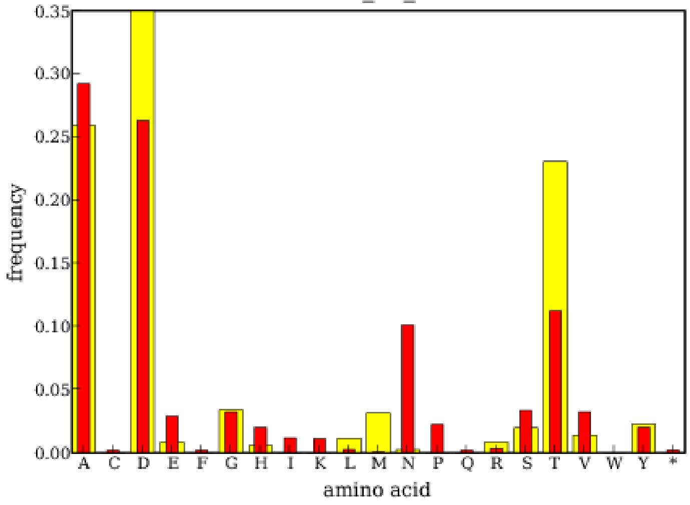

L3_10_5

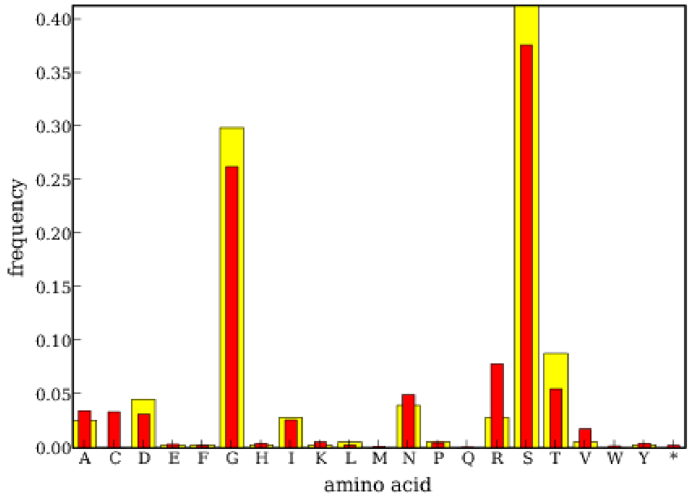

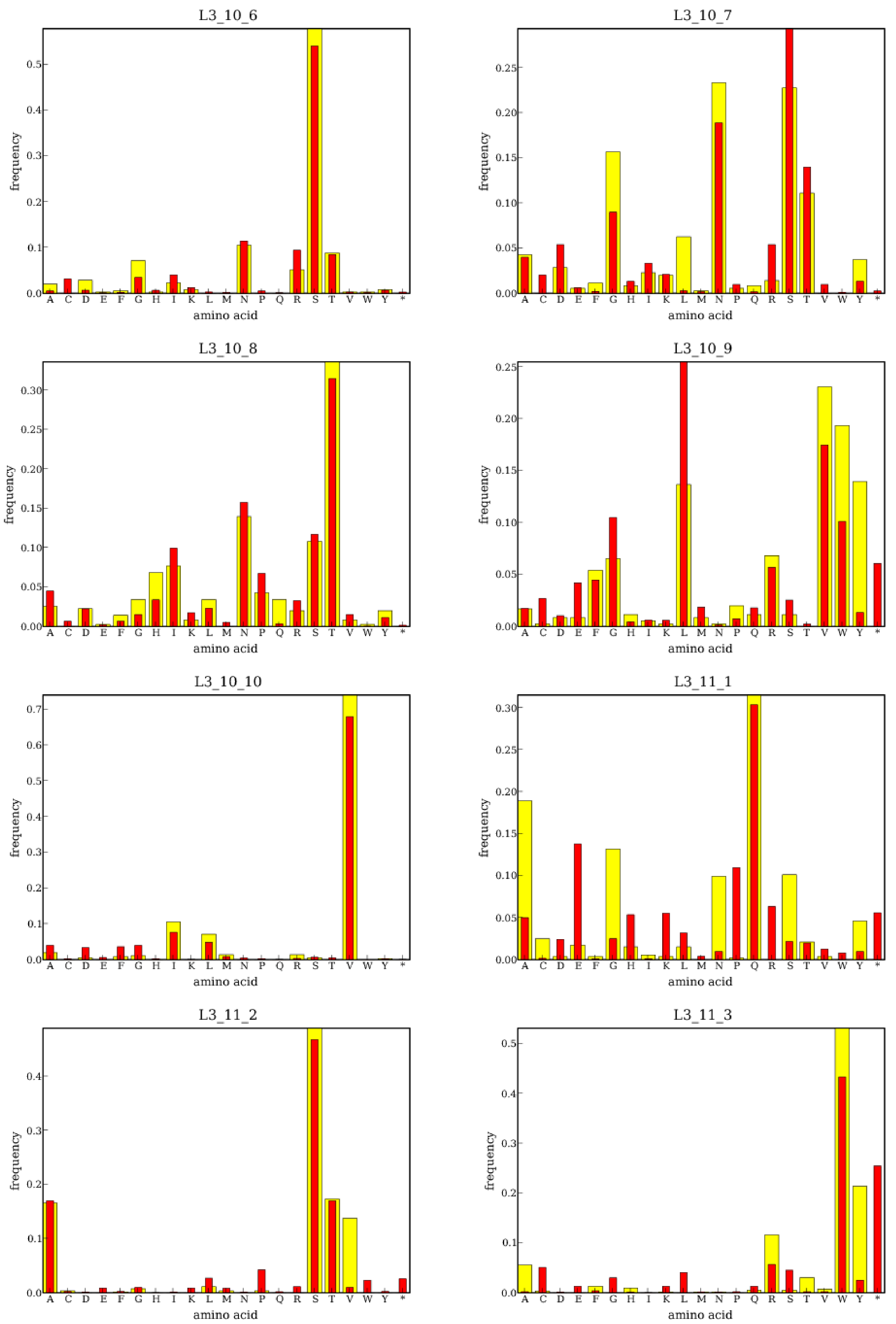
L3_10_6
L3_10_7
L3_10_8
L3_10_9
L3_10_10
L3_11_1
L3_11_2
L3_11_3
frequency
amino acid
A C D E F G H I K L M N P Q R S T V W Y *

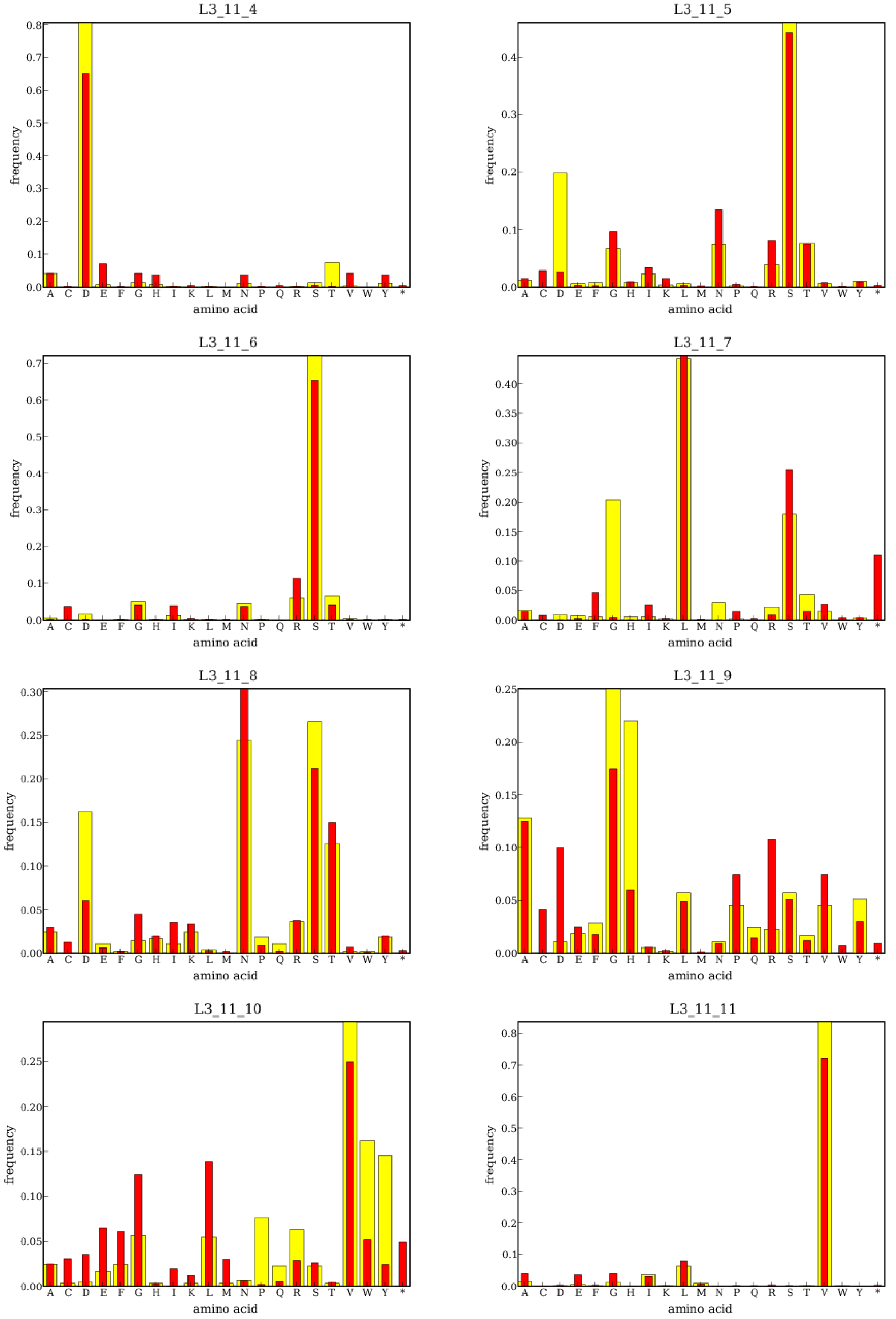

L3_11_4
frequency
amino acid
L3_11_5
frequency
amino acid
L3_11_6
frequency
amino acid
L3_11_7
frequency
amino acid
L3_11_8
frequency
amino acid
L3_11_9
frequency
amino acid
L3_11_10
frequency
amino acid
L3_11_11
frequency
amino acid
