# Supplementary material for: A focused antibody library for selecting scFvs expressed at high levels in the cytoplasm
Source: BMC Biotechnol. 2007 Nov 22;7:81. doi: 10.1186/1472-6750-7-81 (PMC2241821; doi:10.1186/1472-6750-7-81)
Supplement: Additional File 2 — Sequences of the spiked oligonucleotides used to introduce the random CDR3 loops. H3_n = n amino acid long VH CDR3 loop; K3_n = n amino acid long VL κ CDR3 loop; L3_n = n amino acid long VL λ CDR3 loop. For the degenerated positions, the percentages of the 4 bases are given as N(A/C/G/T) [file 1472-6750-7-81-S2.pdf]

Name	Sequence
**H3_5**	AGGGTGCCTCTGCCCCA N(40/5/50/5) N(5/5/5/85) N(60/5/25/10) N(40/10/45/5) N(15/30/5/50) N(5/75/10/10) N(30/35/30/5) N(50/20/10/20) N(60/20/5/15) N(30/25/40/5) N(15/20/25/40) N(35/40/5/20) N(25/50/20/5) N(10/50/10/30) N(5/55/35/5) TCTCACACAGTAATAAACAGCCG
**H3_6**	AGGGTGCCTCTGCCCCA N(55/5/35/5) N(10/5/5/80) N(75/15/5/5) N(45/5/45/5) N(5/10/10/75) N(5/85/5/5) N(45/35/15/5) N(55/20/10/15) N(55/20/10/15) N(40/30/25/5) N(10/40/15/35) N(25/45/5/25) N(45/30/20/5) N(15/40/20/25) N(10/35/30/25) N(20/15/60/5) N(10/60/10/20) N(5/70/10/15) TCTCACACAGTAATAAACAGCCG
**H3_7**	AGGGTGCCTCTGCCCCA N(35/5/55/5) N(15/5/15/65) N(65/15/10/10) N(55/5/35/5) N(5/5/5/85) N(5/85/5/5) N(50/5/40/5) N(75/5/10/10) N(60/10/20/10) N(10/45/40/5) N(15/35/25/25) N(25/55/5/15) N(35/35/25/5) N(10/45/15/30) N(25/45/15/15) N(25/25/25/25) N(20/40/15/25) N(10/35/25/30) N(40/15/40/5) N(5/50/15/30) N(5/80/10/5) TCTCACACAGTAATAAACAGCCG
**H3_8**	AGGGTGCCTCTGCCCCA N(55/5/35/5) N(10/5/10/75) N(75/10/5/10) N(50/5/40/5) N(5/5/5/85) N(5/85/5/5) N(45/5/45/5) N(75/5/10/10) N(65/5/20/10) N(25/45/25/5) N(10/25/40/25) N(30/45/5/20) N(55/10/30/5) N(5/55/15/25) N(5/55/15/25) N(35/35/25/5) N(15/45/20/20) N(15/40/10/35) N(30/40/25/5) N(20/35/20/25) N(15/40/20/25) N(65/5/20/10) N(15/45/5/35) N(5/70/15/10) TCTCACACAGTAATAAACAGCCG
**H3_9**	AGGGTGCCTCTGCCCCA N(40/5/50/5) N(10/5/10/75) N(75/5/15/5) N(40/5/50/5) N(5/5/5/85) N(5/85/5/5) N(45/5/45/5) N(85/5/5/5) N(70/5/15/10) N(55/25/15/5) N(10/25/35/30) N(35/45/15/5) N(10/25/60/5) N(10/35/20/35) N(20/45/15/20) N(30/30/35/5) N(15/40/20/25) N(20/40/15/25) N(65/20/10/5) N(20/40/20/20) N(5/45/15/35) N(15/30/50/5) N(20/45/20/15) N(5/40/25/30) N(30/25/40/5) N(10/40/10/40) N(5/60/20/15) TCTCACACAGTAATAAACAGCCG
**H3_10**	AGGGTGCCTCTGCCCCA N(50/5/40/5) N(20/5/10/65) N(70/10/15/5) N(60/5/30/5) N(5/5/5/85) N(5/85/5/5) N(35/5/55/5) N(85/5/5/5) N(75/5/15/5) N(30/35/30/5) N(10/20/45/25) N(30/45/15/10) N(30/35/30/5) N(5/30/20/45) N(30/40/10/20) N(40/35/20/5) N(15/35/25/25) N(25/35/15/25) N(50/30/15/5) N(10/45/20/25) N(20/50/10/20) N(25/20/50/5) N(20/40/20/20) N(5/50/15/30) N(20/20/40/20) N(20/35/20/25) N(5/35/30/30) N(5/30/60/5) N(15/35/10/40) N(5/60/15/20) TCTCACACAGTAATAAACAGCCG
**H3_11**	AGGGTGCCTCTGCCCCA N(55/5/35/5) N(10/5/10/75) N(80/5/10/5) N(60/5/30/5) N(5/5/5/85) N(5/85/5/5) N(45/5/45/5) N(85/5/5/5) N(75/10/10/5) N(40/30/25/5) N(10/20/35/35) N(35/35/20/10) N(40/30/25/5) N(10/25/20/45) N(35/35/15/15) N(35/30/30/5) N(15/30/25/30) N(35/30/15/20) N(20/35/40/5) N(20/30/25/25) N(30/40/10/20) N(5/35/50/10) N(20/30/30/20) N(30/40/15/15) N(35/35/25/5) N(20/35/20/25) N(20/40/15/25) N(45/25/10/20) N(20/35/20/25) N(20/30/25/25) N(45/25/25/5) N(15/35/10/40) N(5/60/20/15) TCTCACACAGTAATAAACAGCCG
**H3_12**	AGGGTGCCTCTGCCCCA N(45/5/45/5) N(15/5/15/65) N(70/5/15/10) N(40/5/50/5) N(5/5/5/85) N(5/85/5/5) N(45/5/45/5) N(85/5/5/5) N(75/5/15/5) N(10/30/55/5) N(10/30/25/35) N(40/40/15/5) N(25/25/45/5) N(10/25/20/45) N(35/35/10/20) N(40/40/15/5) N(20/20/35/25) N(40/30/15/15) N(10/40/45/5) N(20/25/30/25) N(35/35/15/15) N(30/30/35/5) N(15/40/20/25) N(20/45/5/30) N(15/25/40/20) N(20/30/25/25) N(20/40/15/25) N(15/25/40/20) N(20/35/20/25) N(25/40/15/20) N(50/30/15/5) N(20/45/20/15) N(5/35/35/25) N(45/25/25/5) N(15/35/10/40) N(5/65/15/15) TCTCACACAGTAATAAACAGCCG
**H3_13**	AGGGTGCCTCTGCCCCA N(45/10/40/5) N(15/5/15/65) N(75/5/15/5) N(35/5/55/5) N(5/5/5/85) N(5/85/5/5) N(45/5/45/5) N(85/5/5/5) N(80/5/10/5) N(35/25/35/5) N(10/30/25/35) N(40/45/10/5) N(15/25/55/5) N(10/25/10/55) N(35/35/10/20) N(55/30/10/5) N(15/20/25/40) N(45/25/10/20) N(25/30/40/5) N(20/30/20/30) N(30/35/15/20) N(15/25/55/5) N(15/35/20/30) N(25/45/10/20) N(10/45/40/5) N(20/25/25/30) N(30/40/10/20) N(30/25/30/15) N(20/25/25/30) N(35/35/15/15) N(25/30/30/15) N(20/30/20/30) N(25/35/20/20) N(40/30/25/5) N(20/45/20/15) N(5/35/35/25) N(45/25/25/5) N(10/35/15/40) N(5/65/20/10) TCTCACACAGTAATAAACAGCCG
**H3_14**	AGGGTGCCTCTGCCCCA N(50/5/40/5) N(20/5/15/60) N(60/10/25/5) N(35/5/55/5) N(5/5/5/85) N(5/85/5/5) N(30/20/45/5) N(85/5/5/5) N(80/5/5/10) N(30/35/30/5) N(10/35/20/35) N(40/40/15/5) N(50/25/20/5) N(10/25/10/55) N(40/30/10/20) N(20/20/55/5) N(15/20/20/45) N(45/25/15/15) N(15/25/55/5) N(15/30/25/30) N(35/30/15/20) N(40/30/25/5) N(15/25/25/35) N(35/35/10/20) N(20/30/40/10) N(15/30/25/30) N(25/40/10/25) N(50/25/15/10) N(15/35/25/25) N(35/35/10/20) N(15/25/45/15) N(15/30/25/30) N(35/35/10/20) N(15/30/40/15) N(20/30/20/30) N(30/40/10/20) N(30/30/15/25) N(20/35/25/20) N(20/30/30/20) N(30/25/40/5) N(10/30/15/45) N(5/70/15/10) TCTCACACAGTAATAAACAGCCG
**H3_15**	AGGGTGCCTCTGCCCCA N(30/5/45/20) N(45/5/5/45) N(45/35/5/15) N(45/5/45/5) N(5/5/5/85) N(5/85/5/5) N(45/20/30/5) N(85/5/5/5) N(85/5/5/5) N(20/35/40/5) N(5/40/25/30) N(35/50/10/5) N(15/5/75/5) N(10/10/20/60) N(50/30/10/10) N(25/10/55/10) N(15/20/20/45) N(50/25/15/10) N(35/30/30/5) N(15/25/25/35) N(50/20/15/15) N(35/5/35/25) N(10/30/30/30) N(35/35/10/20) N(20/40/35/5) N(20/20/35/25) N(35/35/15/15) N(45/35/15/5) N(15/30/25/30) N(35/35/10/20) N(35/35/25/5) N(20/30/20/30) N(30/35/10/25) N(30/25/40/5) N(15/30/20/35) N(35/30/15/20) N(35/25/35/5) N(15/35/20/30) N(25/35/25/15) N(5/30/45/20) N(20/35/20/25) N(15/30/30/25) N(45/25/25/5) N(15/35/10/40) N(5/70/15/10) TCTCACACAGTAATAAACAGCCG
**H3_16**	AGGGTGCCTCTGCCCCA N(35/10/30/25) N(65/5/5/25) N(25/55/5/15) N(45/5/45/5) N(5/5/5/85) N(5/85/5/5) N(35/30/30/5) N(85/5/5/5) N(75/5/5/15) N(15/35/45/5) N(5/45/20/30) N(35/55/5/5) N(50/5/40/5) N(10/5/15/70) N(60/20/10/10) N(65/5/25/5) N(10/10/25/55) N(55/15/15/15) N(30/5/60/5) N(10/15/30/45) N(55/15/15/15) N(25/25/45/5) N(20/30/15/35) N(40/35/10/15) N(40/20/30/10) N(20/30/25/25) N(35/30/10/25) N(45/30/15/10) N(15/35/25/25) N(30/35/10/25) N(35/30/25/10) N(20/25/30/25) N(35/35/10/20) N(30/20/30/20) N(20/35/20/25) N(30/40/10/20) N(45/10/25/20) N(20/25/20/35) N(30/35/15/20) N(45/30/20/5) N(15/35/25/25) N(25/35/20/20) N(40/15/5/40) N(20/35/20/25) N(15/30/30/25) N(30/30/35/5) N(15/30/15/40) N(5/75/15/5) TCTCACACAGTAATAAACAGCCG
**H3_17**	AGGGTGCCTCTGCCCCA N(45/10/20/25) N(70/5/5/20) N(15/60/5/20) N(40/5/50/5) N(5/5/5/85) N(5/85/5/5) N(25/20/50/5) N(85/5/5/5) N(80/5/5/10) N(10/45/40/5) N(5/50/20/25) N(30/60/5/5) N(5/5/85/5) N(5/5/15/75) N(65/20/5/10) N(45/5/45/5) N(10/10/20/60) N(55/10/20/15) N(45/5/45/5) N(15/15/20/50) N(60/15/15/10) N(60/20/15/5) N(10/30/20/40) N(45/25/15/15) N(15/35/45/5) N(15/15/35/35) N(40/30/15/15) N(30/25/40/5) N(20/30/25/25) N(30/35/10/25) N(20/30/40/10) N(15/35/25/25) N(30/35/10/25) N(40/30/25/5) N(20/30/25/25) N(40/35/10/15) N(35/20/40/5) N(20/25/20/35) N(40/35/10/15) N(35/20/40/5) N(20/20/20/40) N(35/35/15/15) N(30/20/30/20) N(20/30/20/30) N(25/30/25/20) N(35/35/25/5) N(25/35/20/20) N(5/35/40/20) N(20/30/45/5) N(15/30/10/45) N(5/70/15/10) TCTCACACAGTAATAAACAGCCG
**K3_9**	GGACGAGGCTGATTATTACTGC N(5/85/5/5) N(85/5/5/5) N(5/5/85/5) N(5/85/5/5) N(85/5/5/5) N(40/5/50/5) N(5/5/10/80) N(65/25/5/5) N(5/50/5/40) N(30/5/25/40) N(60/10/20/10) N(5/40/10/45) N(80/5/10/5) N(30/15/50/5) N(5/60/5/30) N(35/5/10/50) N(30/40/10/20) N(5/15/45/35) N(5/85/5/5) N(5/85/5/5) N(5/5/85/5) N(5/40/5/50) N(30/10/25/35) N(5/35/50/10) N(85/5/5/5) N(5/85/5/5) N(5/50/30/15) TTCGGCGGAGGGACCAAG
**K3_10**	GGACGAGGCTGATTATTACTGC N(5/85/5/5) N(85/5/5/5) N(5/5/85/5) N(5/85/5/5) N(85/5/5/5) N(40/5/50/5) N(5/5/5/85) N(80/10/5/5) N(25/20/5/50) N(35/5/40/20) N(45/5/45/5) N(5/30/15/50) N(75/10/10/5) N(40/5/50/5) N(5/45/5/45) N(20/5/5/70) N(10/45/40/5) N(5/5/85/5) N(5/85/5/5) N(5/85/5/5) N(5/5/85/5) N(5/85/5/5) N(5/65/15/15) N(5/5/85/5) N(15/5/10/70) N(20/5/30/45) N(5/30/50/15) N(85/5/5/5) N(5/85/5/5) N(5/40/5/50) TTCGGCGGAGGGACCAAG
**L3_9**	GGACGAGGCTGATTATTACTGC N(5/85/5/5) N(80/5/5/10) N(5/5/85/5) N(20/5/30/45) N(5/75/5/15) N(85/5/5/5) N(5/5/5/85) N(35/5/55/5) N(5/10/80/5) N(5/10/80/5) N(80/10/5/5) N(5/60/5/30) N(65/5/25/5) N(15/10/70/5) N(5/65/5/25) N(70/5/20/5) N(20/10/65/5) N(5/60/5/30) N(65/10/20/5) N(15/45/20/20) N(5/40/10/45) N(15/5/50/30) N(10/15/25/50) N(5/20/70/5) N(10/5/80/5) N(5/5/5/85) N(5/40/10/45) TTCGGCGGAGGGACCAAG
**L3_10**	GGACGAGGCTGATTATTACTGC N(45/5/10/40) N(15/15/60/10) N(10/50/5/35) N(5/5/5/85) N(5/85/5/5) N(45/5/45/5) N(5/5/5/85) N(85/5/5/5) N(5/50/5/40) N(25/5/65/5) N(45/45/5/5) N(5/45/5/45) N(55/5/35/5) N(10/10/75/5) N(5/40/5/50) N(85/5/5/5) N(15/10/70/5) N(5/50/5/40) N(70/5/20/5) N(30/20/45/5) N(5/75/5/15) N(70/15/10/5) N(25/45/15/15) N(5/35/5/55) N(5/15/35/45) N(15/5/30/50) N(5/15/75/5) N(10/5/80/5) N(5/5/5/85) N(5/50/10/35) TTCGGCGGAGGGACCAAG
**L3_11**	GGACGAGGCTGATTATTACTGC N(10/55/25/10) N(65/20/10/5) N(5/10/80/5) N(20/5/20/55) N(5/85/5/5) N(5/5/85/5) N(5/5/5/85) N(30/5/60/5) N(5/5/85/5) N(5/5/85/5) N(85/5/5/5) N(5/45/5/45) N(75/5/15/5) N(20/10/65/5) N(5/60/5/30) N(85/5/5/5) N(5/5/85/5) N(5/35/5/55) N(5/5/5/85) N(5/30/10/55) N(85/5/5/5) N(75/5/15/5) N(45/20/30/5) N(5/20/5/70) N(5/30/50/15) N(25/25/35/15) N(5/45/15/35) N(10/5/50/35) N(20/5/25/50) N(5/20/60/15) N(5/5/85/5) N(5/5/5/85) N(70/5/20/5) TTCGGCGGAGGGACCAAG
