# Supplementary material for: A focused antibody library for selecting scFvs expressed at high levels in the cytoplasm
Source: BMC Biotechnol. 2007 Nov 22;7:81. doi: 10.1186/1472-6750-7-81 (PMC2241821; doi:10.1186/1472-6750-7-81)
Supplement: Additional File 3 — Sequence of randomly picked clones. Sequences of 118 scFvs randomly picked in the library. For some clones the sequence quality was not good enough to read the light chain CDR3 and are noted nr (non-read). [file 1472-6750-7-81-S3.pdf]

name	Heavy CDR3	Light CDR3
scFv1	GSERLDC	EQSKNLPST
scFv2	SSITIFGGGMDF	nr
scFv3	GRIEIDC	QHSGYWPPFT
scFv4	RVCTIYTSGFDC	RQDINSSPLT
scFv5	GVVLWRELEF	QAKDGSLISLV
scFv6	VDDEGTIYWSFGY	HHSNNKPWT
scFv9	AVLELNY	QQSASKPWT
scFv11	EVGCGKNWWLDG	QQSYDLPQT
scFv13	SSITIFGGGMDI	SSSASRIHFA
scFv15	AVHFGWEWGYVDY	HTGVDSSSSVV
scFv16	ELEGFDN	QHYSRSTPCT
scFv17	NRDVGLIYYCLDY	QHYRSHPYT
scFv18	SPKNTWELNK	LNSLHRTGL
scFv19	EGGEHWVFDS	ETCDSILSGEE
scFv20	GRVACFVFIDLIYY	QKYSNFPCT
scFv21	SSITIFGGGMDV	HQLCNTLAFT
scFv23	GGYFWATPAFES	HQYFRSPLST
scFv24	CWGAGQCDKFGLHY	QQYDITSQT
scFv25	GFGGEFDY	QQSCNEPLT
scFv27	NGLISVGWAVDN	QRYSSTPLT
scFv28	GEGRHGY	CSRTSSNIAV
scFv31	AISSQKDCNY	QQYDKITWT
scFv32	DGDGRCNIDD	YSYIHSTIGV
scFv33	SSITIFGGGMDD	KAFNKDNMV
scFv34	GIGGCELFVY	nr
scFv35	SVAGFTF	QQNNNYPGT
scFv36	ELSSAESRDY	QQFQSVSSY
scFv37	SSITIFGGGMDA	PQYKHWPLST
scFv38	RSGDDAFFDV	QTRHNTLIRMT
scFv41	DRRGVFDD	QHFHAPPLT
scFv44	SSITIFGGGMDV	AVSDSRAKV
scFv45	DRPHRFDN	QHYHTIPFT
scFv47	SSITIFGGGMDV	LSLNGSNIV
scFv50	ESVYYYDDGGCDTELEV	QASDRSHLV
B4	KHFDDEGLLIYQEACEA	SRYDSRNTAN
B5	RSDDWDGVDD	PLFDSSPAHT
B6	GGTGCFFVY	QQKIAGVVP
B7	SSITIFGGGMDD	QSRKRSSDPLL
B8	WPRADYWNVCDY	HSVPCSPVF
B9	DPGHSKHFFF	QQYLLSPPHT
B10	YCLIY	QSWRDNIMI
B13	IKGASARLVYTLGMF	nr
B14	HRIGSGVHGYLDF	nr
B15	SGEYNCLFVNSFNI	RSYKGRCNSV
B16	ERFDGVCCNDFDS	nr
B17	DSTRHLVM	HQYYTLMST
B19	YPAVGRDSSGSYTWFED	LSSHSGTSL
B21	DGVLRFETDGSHDVVDI	YHYVNWPLFT
B23	QSWHGIFFL	nr
B24	GSGGIHY	QQKNSLPHT
B26	GGRCVGENSFDS	LVCGIGTVV
B27	VGGAFAG	EKGHTNTVG
B28	DACNHKNSRGDKDWFDI	SSNDGKTNVV
B29	CGGSEFDF	HQYSNSPRLT
B30	QTFDNIAVV	nr
B31	VGRGSAELGY	nr
B33	AESEGATLVF	ITSITSIISV
B34	WGVDCTALEH	HACRGVTAV
B35	VDALDCCDY	nr
B36	GGDLSESRIESEFWLDV	QQSNNNRVT
B37	HDMLIGCRSLFDY	QHYHTWPTCT
B38	DLGRLFDY	PSWDTSVSVCL
B39	AHGKNGSRPEYSLDV	QQFHSNILV
B41	SSITIFGGGNDD	QQSDNILNRLE
B42	GVAGSFQD	QSWHSANLV
B43	QARYTSLDVLL	nr
B44	PTKGDYGFDQ	EKDYDSQLTVT
B47	DPGKKACCMCAWLDL	QAYDSXTRI
B48	VNCSGEALDH	PLLDISILV
B53	SSITIFGGGMDI	KLEDITAST
B54	DHERVCYGLEDELDR	HTFNCTTSI
B56	GRRLVHGKDFDY	ETTDSHVFR
B57	GFHGEFWALSFRWDL	QSNTRRPTLVM
B58	QQLLSPWVT	nr
B59	SGSSDYFEY	QQYDNSSYT
BA14	ELQCTNGFNY	SYDTSNPFF
BA15	CVSVLGETHSYDY	MSWATTSSV
BA18	SNINIFGGGMDD	QTGDTNLKVLV
BA19	DHAYSQYGVYVEFDD	QTCDGNIVF
BA20	HQHGHKPLGT	nr
BA21	GTIGVVDGGWFSY	QQYYNSSLYH
BA22	KAPPNSYCFAV	QPLDSHPPYT
BA23	LINDWFDN	nr
BA24	GTGDKRFYDYYFDY	PPDTNAPPLT
BA25	GDVDIDL	QQYRNSSPLT
BA26	EFFGRAVSAVRSDY	QQYSLSPPYT
BA27	AVGGGRPFDY	QSNYTARFV
BA28	SSITIFGGGMDV	QKSCNAPLLT
BA29	EQDNHSPLVT	nr
BA30	PGPSIFCCKSDYGFDV	RSHDGSIPXV
BA31	GIQFMAVMNGFDY	PHYDILLPST
BA32	IVLPVVDY	RQSNNSLPFT
BA35	EHGHDMCCGFDN	QLYCNTQPDN
BA36	GDCGTADY	HQSDTSPALT
BA37	VREQMMQSSTLSLFS	LQYKNTRLCT
BA39	SSRYMADGEDYKFLFDG	LSWAGHTVA
BA40	PSITIFGGGMDV	QQYPSCPLNT
BA41	GLEYGARFSYVAFDT	nr
BA42	EQYNSSQPIT	nr
BA43	DHGTECCDCYDAFDV	QQHYNNSRLT
BA44	GRWGVDF	HHYHTLQPFT
BA46	HGGSRFDY	nr
BA48	VLKLTPEYAYCFGY	nr
BA50	DSNAY	nr
BA51	DWSAHNGSRLDY	nr
BA52	EAWSVSYFCNCFDL	nr
BA53	VSKGNADH	nr
BA54	EKALTWSFDD	nr
BA56	YSSVEVDKNGFDY	nr
BA57	EDSWTCNFDY	nr
BA58	DRNFTSGDYKCYWL	nr
BA61	GTSWGSGS	nr
BA62	GGRIGACYNDDVDYL	nr
BA63	DRGRFDE	nr
BA66	GACDFDV	nr
BA67	AVLFSNWSCGLAL	nr
BA68	DREHLDDGCEFDG	nr
BA69	SSITIFGGGMDV	nr
