# Supplementary material for: A focused antibody library for selecting scFvs expressed at high levels in the cytoplasm
Source: BMC Biotechnol. 2007 Nov 22;7:81. doi: 10.1186/1472-6750-7-81 (PMC2241821; doi:10.1186/1472-6750-7-81)

A

	CDR-H3	CDR-L3
**Clone 2**	ASRGLHWRRRFDY	RLWDSGTVI
**Clone 5**	RRSGRFVY	QASDRSTSV
**Clone 6**	RSRRGSVY	ESSATSSHRLL
**Clone 9**	DRRRKFDY	HQKTGSPST
**Clone 10**	RRSRVNYGRLDY	QQHDRKPHT

B

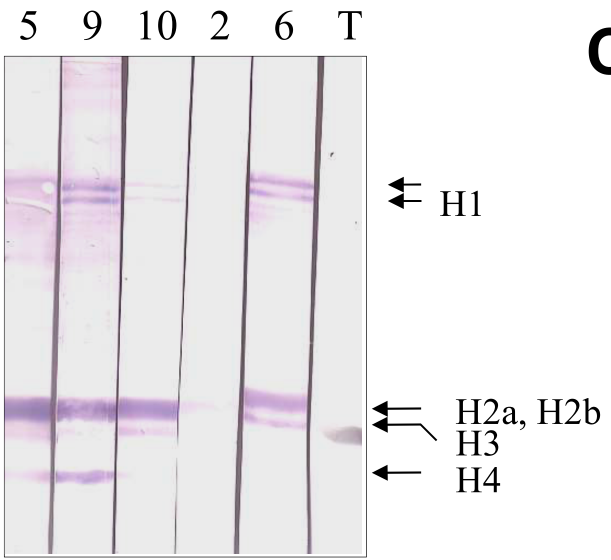

C

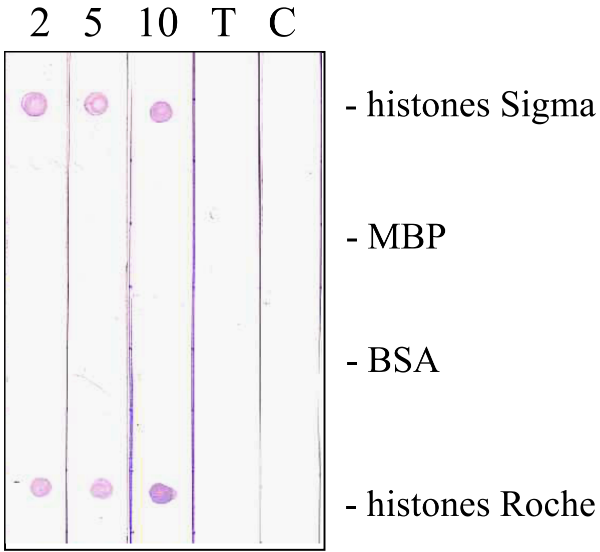

Supplement: Additional File 4 — In vitro characterization of some anti-histones scFvs. In cell screened anti-histones (Figure 6) were expressed and purified from E. coli. (A) sequence of the clones. (B) reactivity measured by western blot and (C) dot blot. The histones preparation used for the selection was from Sigma. "histones Roche" is another histones preparation obtained from Roche. MBP: Maltose Binding Protein. T: tubulin clone 2F12C (Table 2). C: negative control (no scFv). The sequences of the clones 2, 5, 6, 9 and 10 have been deposited in the EMBL database and their accession numbers are respectively AM888346AM888346, AM888347, AM888348, AM888349 and AM888350AM888350. [file 1472-6750-7-81-S4.pdf]
