# Supplementary figures and images for: A focused antibody library for selecting scFvs expressed at high levels in the cytoplasm
Source: BMC Biotechnol. 2007 Nov 22;7:81. doi: 10.1186/1472-6750-7-81 (PMC2241821; doi:10.1186/1472-6750-7-81)

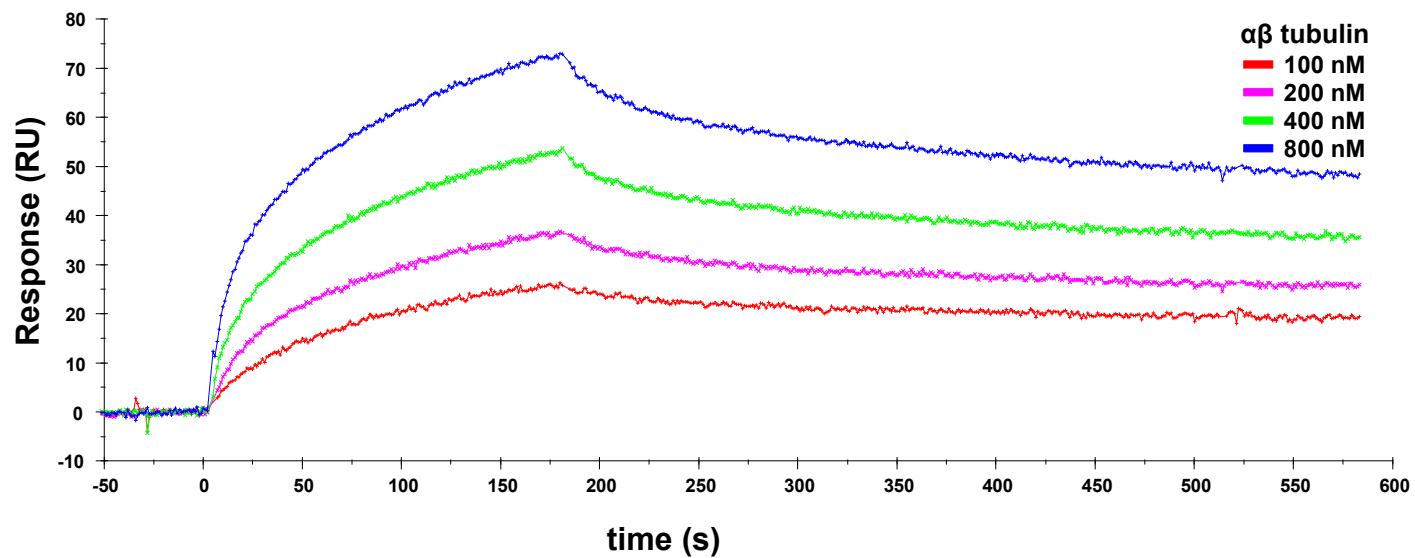

Supplement: Additional File 5 — Biacore analysis of clone 2F12C. ScFv affinity was determined on a BIACORE 2000 apparatus (Biacore AB, Uppsala, Sweden). HBS-EP is 0.01 M HEPES, pH 7.4, 0.15 M NaCl, 3 mM EDTA, 0.005% P20 surfactant. Anti-myc antibody (purified 9E10 from Sigma) was covalently immobilized on a flow cell of a carboxymethyl dextran sensorchip (CM5 from Biacore AB) using the amine coupling method according to the manufacturer's instructions. The immobilization level was around 4000 resonance units (RU). A second flowcell was treated with the same chemical procedure without the 9E10 and used as a reference. After the injection of the scFv (50 μg/ml in HBS-EP containing 0.1% dextran), different concentrations of tubulin in HBS-EP were injected during 180 s over the two flowcells and followed by a dissociation step of 400s. The experiments were performed at a 50 μl/min flow rate at 25°C. Between each run, sensor surfaces were regenerated with a pulse of 25 mM HCl. All the sensorgrams were corrected by subtracting the signal from the reference flowcell and were globally fitted using BIAevaluation version 3.2 software (Biacore AB) to a two-state reaction model (A+B←kd1→ka1AB←kd2→ka2AB∗ MathType@MTEF@5@5@+=feaafiart1ev1aaatCvAUfKttLearuWrP9MDH5MBPbIqV92AaeXatLxBI9gBaebbnrfifHhDYfgasaacPC6xNi=xH8viVGI8Gi=hEeeu0xXdbba9frFj0xb9qqpG0dXdb9aspeI8k8fiI+fsY=rqGqVepae9pg0db9vqaiVgFr0xfr=xfr=xc9adbaqaaeGacaGaaiaabeqaaeqabiWaaaGcbaGaemyqaeKaey4kaSIaemOqai0aa0baaSqaamaaoGbameqabaGaem4AaSMaemizaqMaeGymaedaliaawcziaaqaamaaoqcameaacqWGRbWAcqWGHbqycqaIXaqmaeqaliaawkziaaaakiabdgeabjabdkeacnaaDaaaleaadaGdyaadbeqaaiabdUgaRjabdsgaKjabikdaYaWccaGLqgcaaeaadaGdKaadbaGaem4AaSMaemyyaeMaeGOmaidabeWccaGLsgcaaaGccqWGbbqqcqWGcbGqdaahaaWcbeqaaiabgEHiQaaaaaa@494B@), where ka1 and kd1 are the association and dissociation rate constants for the first equilibrium, and ka2 and kd2 for the second. ka1 = (4.39 ± 0.06) 104 M-1 s-1; kd1 = (3,65 ± 0,10) 10-2 s-1; ka2 = (1.37 ± 0.0178) 10-2 s-1; kd2 = (8.03 ± 0.113) 10-4 s-1; Kd = ( [file 1472-6750-7-81-S5.pdf]
